# Non-fluorinated non-solvating cosolvent enabling superior performance of lithium metal negative electrode battery

**DOI:** 10.1038/s41467-022-32192-5

**Published:** 2022-08-04

**Authors:** Junyeob Moon, Dong Ok Kim, Lieven Bekaert, Munsoo Song, Jinkyu Chung, Danwon Lee, Annick Hubin, Jongwoo Lim

**Affiliations:** 1 Department of Chemistry, College of Science, Seoul National University, Seoul, 08826 Republic of Korea; 2 Department of Chemistry and Biochemistry, Swarthmore College, Swarthmore, PA 19081 USA; 3 Vrije Universiteit Brussel, Research Group Electrochemical and Surface Engineering, Pleinlaan 2, 1050 Brussels, Belgium; 4 Molecular Science Research Institute (MSRI), Seoul National University, Seoul, 08826 Republic of Korea

**Keywords:** Energy, Batteries

## Abstract

The growth of dendrites on lithium metal electrodes is problematic because it causes irreversible capacity loss and safety hazards. Localised high-concentration electrolytes (LHCEs) can form a mechanically stable solid-electrolyte interphase and prevent uneven growth of lithium metal. However, the optimal physicochemical properties of LHCEs have not been clearly determined which limits the choice to fluorinated non-solvating cosolvents (FNSCs). Also, FNSCs in LHCEs raise environmental concerns, are costly, and may cause low cathodic stability owing to their low lowest unoccupied molecular orbital level, leading to unsatisfactory cycle life. Here, we spectroscopically measured the Li^+^ solvation ability and miscibility of candidate non-fluorinated non-solvating cosolvents (NFNSCs) and identified the suitable physicochemical properties for non-solvating cosolvents. Using our design principle, we proposed NFNSCs that deliver a coulombic efficiency up to 99.0% over 1400 cycles. NMR spectra revealed that the designed NFNSCs were highly stable in electrolytes during extended cycles. In addition, solvation structure analysis by Raman spectroscopy and theoretical calculation of Li^+^ binding energy suggested that the low ability of these NFNSCs to solvate Li^+^ originates from the aromatic ring that allows delocalisation of electron pairs on the oxygen atom.

## Abstract (web summary)

Localised high-concentration electrolyte is key to prevent uneven growth of lithium metal by forming a mechanically stable solid-electrolyte interphase. Here, the authors identify the suitable physicochemical properties for non-solvating co-solvents that improve the performance of lithium metal battery.

## Introduction

Long-lasting electric vehicles require batteries with higher energy densities than conventional lithium-ion batteries (LIB)^1^. Researchers in the LIB industry are now paying special attention to the lithium metal electrode (LME)^1–3^ owing to its high energy density (3860 mAh g^–1^) and low electrochemical potential (–3.04 V vs. the standard hydrogen electrode)^4^. However, the dendritic growth of lithium metal, which leads to battery failure, remains a problem^5^. A solid-electrolyte interphase (SEI) having high mechanical strength was reported to be effective in preventing dendrite formation^6,7^. Such an SEI layer stops unwanted reactions between the electrolytes and lithium metal and prevents dendrite growth to conserve the lithium metal source. Thus, the formed SEI layer should be highly resilient and uniform to prevent continuous dendritic growth. Recent studies have demonstrated that localised high-concentration electrolytes (LHCEs), created by adding a non-solvating cosolvent, can induce compact lithium-ion–anion solvation pairs that help produce a strong inorganic SEI^8–13^. Because the LHCE has a lower viscosity and higher lithium ion conductivity than the highly concentrated electrolyte (HCE), it has better wettability on the electrodes and separators under practical conditions^11^.

Unfortunately, there is a lack of design strategies for ideal non-solvating cosolvents, and the reported cases are limited to fluorinated non-solvating cosolvents (FNSCs)^14–16^. The highly electronegative fluorine withdraws electrons from oxygen in adjacent FNSC molecules, resulting in a lower solubility of the lithium ion^17^. Further, fluorination decreases the cathodic stability, resulting in facile decomposition of FNSCs to form a LiF-rich SEI that is beneficial for LME cycling^11,18,19^. Thus, many researches on LHCEs have focused on using FNSCs such as bis(2,2,2-trifluoroethyl) ether (BTFE), 1,1,2,2-tetrafluoroethyl-2,2,3,3-tetrafluoropropyl ether (TTE), tris(2,2,2-trifluoroethyl)orthoformate (TFEO), fluorobenzene (FB), 1,2-difluorobenzene (DFB) and bis (2,2-difluoroethyl) ether (BDE)^8,11,14,15,20,21^. However, a largely overlooked issue is the accelerated decomposition of cosolvent to produce the SEI, which leads to electrolyte dry-up and ultimately battery failure^22–25^. Furthermore, the high cost and potential environmental hazards of FNSCs necessitate the development of non-fluorinated non-solvating cosolvents (NFNSCs)^15,26,27^.

In this study, we present a design rule for the ideal NFNSCs, based on the superior cycling performance of some candidate compounds over 350 cycles (99.0%, ethoxybenzene), 500 cycles (98.5%, anisole (AN)), and 1400 cycles (99.0%, furan). The lithium-ion solvation ability and miscibility of solvents were experimentally characterised to identify desirable physicochemical properties of the non-solvating cosolvents. Resonance of an electron pair on the oxygen atom of NFNSC molecules decreases the lithium-ion solvation ability, thereby achieving desirable non-solvating characteristics while maintaining good miscibility, superior cathodic stability, and low price.

## Results

### Physicochemical properties of NFNSCs and their design strategy

A non-solvating cosolvent, also known as a diluent, maintains the beneficial solvation structure in an HCE while lowering the viscosity and production cost for applications in conventional cell geometries^28,29^. Due to a lack of solvent molecules solvating lithium ions, both HCE and LHCE contain the solvation structures of lithium-ion–anion pairs, namely the contact ion pair (CIP) and aggregate (AGG). The anion-rich solvation structure further generates an SEI containing abundant inorganic phases, resulting in a higher mechanical durability for stabilising the lithium metal negative electrode interface.

**Fig. 1:**
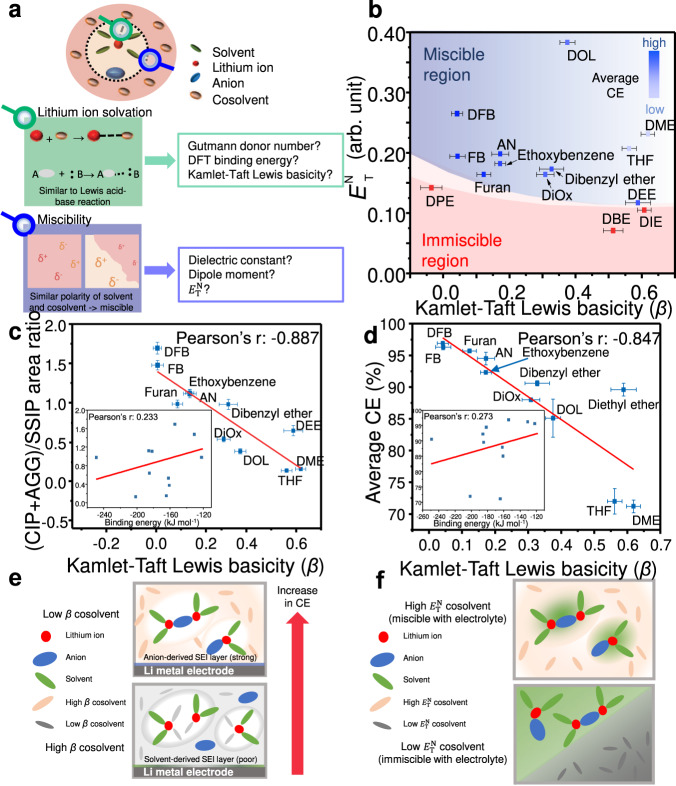
Considerations in the design of cosolvents for LHCEs. **a** Schematic illustration of the parameters for determining non-solvating cosolvents. **b** Two-dimensional plot of the Kamlet-Taft Lewis basicity (β) vs. normalised molar electronic transition energy (${E}_{T}^{N}$) for each solvent. **c** Correlation between the Raman deconvolution result and β. Inset: correlation between the Raman deconvolution result and calculated binding energy of a lithium ion in the gas phase (vacuum condition). **d** Correlation between the average coulombic efficiency (CE) and β. The average CE was obtained from the Li|Cu coin cell using the Aurbach method. Inset: correlation between the average CE and calculated binding energy of a lithium ion in the gas phase (vacuum condition). **e** Schematic of the correlation between β and the performance (CE) of lithium metal batteries employing different electrolytes. **f** Schematic of the correlation between ${E}_{T}^{N}$ and the miscibility of electrolytes for lithium metal battery. Error bars shown here are 95% confidential interval (CI).

As mentioned earlier, adding non-solvating cosolvents to form the LHCE structure is a promising strategy because it can effectively lower the viscosity and cost of HCE. However, the design of ideal non-solvating cosolvents remains challenging because multiple components need to be considered simultaneously. The non-solvating cosolvents must not coordinate with lithium ions or react with the lithium metal negative electrode, so as to preserve the local solvation shell of HCE while staying miscible with the solvating solvent^30^. Conventionally, the lithium-ion solvation ability and miscibility of a (co)solvent may be predicted using its physicochemical parameters such as the dielectric constant, dipole moment, and calculated binding energy between a lithium ion and the (co)solvent molecule (Fig. 1a)^14,16,31,32^. Nevertheless, a cosolvent mixed with several other components in the electrolyte might behave differently from its pure form^33^. The binding energy of a lithium ion calculated by density functional theory (DFT) is limited to local interactions (between single lithium ion and single solvent molecule) and obtained under vacuum condition; thus it is less accurate for predicting the complex solvation energetics in real electrolytes (Fig. 1c, d, inset)^34^. The dipole moment and dielectric constant, which only capture the polarity properties of the pure component, also fail to represent either the lithium-ion solvation ability or the miscibility of the cosolvent in an ensemble with a solvating solvent and concentrated salts (Supplementary Fig. 1c, d)^32,35^.

For example, the dielectric constant and dipole moment of tetrahydrofuran are 7.6 and 1.63 D, while those of 1,2-difluorobenzene are 14.26 and 2.53 D, respectively. Despite the higher values, 1,2-difluorobenzene has a lower ability to solvate lithium ion compared to tetrahydrofuran^14,36^. Thus, we need a new set of solvent descriptors that consider the mutual interactions with other components in the electrolyte for designing new classes of cosolvents^35,37,38^. We propose experimental methods to spectroscopically characterise the lithium-ion solvation ability and miscibility of a cosolvent with other polar species in the electrolyte. A total of 14 model cosolvents were considered to verify the physicochemical parameters of our choice: anisole (AN), ethoxybenzene, diphenyl ether (DPE), dibenzyl ether, fluorobenzene (FB), furan, diethyl ether (DEE), 1,2-dimethoyethane (DME), diisopropyl ether (DIE), tetrahydrofuran (THF), dibutyl ether (DBE), 1,4-dioxane (DiOx), 1,2-difluorobenzene (DFB), and 1,3-dioxolane (DOL) (Supplementary Fig. S2). Each of the 14 model cosolvents was placed in 1 M_solv_ lithium bis(trifluoromethanesulfonyl)imide (LiTFSI) in ethylene carbonate (EC): diethyl carbonate (DEC) (1:1 v/v). Total volume ratio between EC:DEC:cosolvent was set to 1:1:4. The solvation of lithium ion was examined by measuring the Raman spectra, and the performance of electrolyte was measure through average coulombic efficiency (CE), and cycling behaviour in Li|Cu coin cells. The miscibility of electrolytes and solvent mixtures was visually examined (Supplementary Fig. 1a). Note that M_solv_ represents the moles of salt per litre of solvent^39–41^.

It is imperative to measure the stabilisation energy of a lithium ion in a specific solvation structure to assess the solvation ability. This stabilisation energy is defined as the energy difference between the solvated and non-solvated states of the lithium ion. To mimic this definition, we utilised two dyes that form solvation structures with the solvent of interest and measured the degree of dye stabilisation. One dye contains a primary amine group (–NH_2_) that forms a hydrogen bond with the surrounding solvent molecules, whereas the other dye with the tertiary amine group (–N(CH_2_CH_3_)_2_) does not form a hydrogen bond. The two dyes exhibit specific UV-Vis absorption wavelengths depending on the type of interaction with the solvent. The difference between the absorption energies of the two dyes represents their stabilisation energies when solvated in a solvent through hydrogen bonding. The solvation environment around the hydrogen atom of the primary amine group may be equivalent to that around the lithium ion. This way, the stabilisation energy of lithium ions in a specific solvent environment could be estimated. The strong interaction between electrons of the solvent molecules and lithium ion is analogous to the Lewis acid-base interaction; therefore, we refer to the solvation ability of the solvent as the Kamlet-Taft Lewis basicity (*β*) (Fig. 1a)^42^. The method is as follows (Supplementary Fig. 3).

1. The absorption energy of a dye with –NH_2_ group (4-nitroaniline, NA) in the solvent was measured. This energy corresponds to the Lewis acid-base interaction energy between the N-H group in NA and the solvent, plus the additional van der Waals interaction.
2. The absorption energy of another dye with –N(CH_2_CH_3_)_2_ group (*N*,*N*-diethyl-4-nitroaniline, DA) in the solvent was also measured. This energy corresponds to the van der Waals interaction between DA and the solvent.
3. The absorption energy in (2) was fitted to a predetermined linear calibration curve of absorption energies between DA and NA in a nonpolar solvent, in order to simulate the absorption energy of NA in the absence of acid-base interactions. This allows isolation of the van der Waals interaction effect.
4. The difference between the absorption energies measured in steps (1) and (3) gives *β*, which reflects the acid-base interaction between NA and the solvent while completely removing the van der Waals effect. For the details on calculation, refer to Supplementary Note 2^42,43^.

Here, we show that *β* is highly correlated with the solvation structures characterised by Raman spectroscopy, a widely used tool to assess the degree of lithium ion–anion–solvent coordination (Supplementary Fig. 4). A lower *β* value of the cosolvent raises the ratio of AGG and CIP to solvent-separated ion pairs (SSIP), (CIP + AGG)/SSIP. Higher AGG and CIP ratio over SSIP is the ideal geometry for LHCE (Fig. 1c, Supplementary Fig. 4b)^44^. The correlation degree of *β* with (CIP + AGG)/SSIP (Pearson’s r: –0.887) was higher than that of the calculated binding energy of a lithium ion with (CIP + AGG)/SSIP (Fig. 1c, Supplementary Fig. 5b). This conclusion applies to the calculated binding energy of a lithium ion under both vacuum (Pearson’s r: 0.233) and solvent models (EC:DEC = 1:1 v/v, Pearson’s r: 0.771).

The performance of the lithium metal negative electrode was evaluated using the modified Aurbach method to measure the average CE of the cell (for details see the “Methods” section). The Pearson’s r value confirmed that the average CE is more highly correlated with *β* (Pearson’s r: –0.847) than the calculated binding energy of a lithium ion under a vacuum model (Pearson’s r: 0.273). This proves that *β* value of the cosolvent is a critical descriptor for the performance of the lithium metal negative electrode (Fig. 1d). The binding energy of a lithium ion, as well as the associated correlation, was recalculated in an implicit solvent model (EC:DEC = 1:1 v/v). The correlation between the average CE and the recalculated binding energy of a lithium ion (solvent model) is lower (Pearson’s r: 0.751) than that with *β* (Supplementary Fig. 5a).

We stated earlier that the ability of a solvent to solvate lithium ion is closely related to its Lewis basicity (Fig. 1a). As a widely adopted parameter describing the Lewis basicity, the Gutmann donor number demonstrates a lower correlation with the average CE (Pearson’s r: −0.728), which supports that *β* is a better Lewis basicity parameter (Supplementary Note 1, Supplementary Fig. 1b). This result can be attributed to the Lewis acid (SbCl_5_), which is considerably larger in size than that of the lithium ion, used to calculate the Gutmann donor number^28^. Van der Waals radius of the lithium ion is 1.82 Å whereas the shorter Sb-Cl bond length of SbCl_5_ is 2.33 Å^45^. To the best of our knowledge, such an exclusive effect of *β* on the solvation structure and battery performance, especially the average CE, has not been established before (Fig. 1e). Other than the average CE measured using the modified Aurbach method, we also observed the cycling performance of Li|Cu cells using the electrolytes studied in Fig. 1 (Supplementary Fig. 8). The retention of CE during cycling roughly, but not exactly, follows the *β* value of the cosolvents used in the electrolyte system.

We propose another solvatochromic parameter, ${E}_{{{{{{\rm{T}}}}}}}^{{{{{{\rm{N}}}}}}}$ (normalised molar electronic transition energy), which was previously determined by Christian Reichardt as a descriptor for the miscibility of a solvent^46^. It can directly measure the stabilisation energy of a solvent in a highly polar electrolyte due to the intermolecular attraction among the components. The stabilisation energy of a solvent used to calculate ${E}_{{{{{{\rm{T}}}}}}}^{{{{{{\rm{N}}}}}}}$ is the difference between the energies of this solvent molecule in the isolated state and when mixed in an electrolyte. Because the miscibility of a solvent is best defined by the mixing enthalpies of species in the ensemble, the experimentally characterised intermolecular forces between two dissimilar species (one is the solvent of interest and the other is a polar substance representing the highly polar environment of the electrolyte) represent the miscibility of a solvent better than the dielectric constant and dipole moment of the pure species (Fig. 1a, Supplementary Fig. 1c, d, Supplementary Note 3).

Although parameters such as the dielectric constant and dipole moment guide the miscibility between dissimilar molecules based on conventional wisdom (i.e., like-dissolves-like), they are clearly limited when it comes to the precise determination of miscibility (Supplementary Fig. 1c, d). Meanwhile, ${E}_{{{{{{\rm{T}}}}}}}^{{{{{{\rm{N}}}}}}}$ measures the absorption energy of 2,6-diphenyl-4-(2,4,6-triphenyl-1-pyridino) phenolate (Reichardt’s dye) when it is mixed in a solvent of our interest^43,46^. The ground state of the dye becomes more stabilised in a polar solvent because it is zwitter-ionic species and its anionic phenolate part attracts the solvent which has partial positive charge. Eventually, the absorption energy of the dye increases as the polarity of the solvent increases (Supplementary Fig. 7)^47^. Thus, solvents with a high ${E}_{{{{{{\rm{T}}}}}}}^{{{{{{\rm{N}}}}}}}$ should experience a strong attraction to a highly polar electrolyte, that is, miscibility within the system (Fig. 1b, f).

Based on the above considerations, the two physicochemical parameters *β* and ${E}_{{{{{{\rm{T}}}}}}}^{{{{{{\rm{N}}}}}}}$ were determined for a conventional FNSC to assess the applicability of the parameters or our choice (*β*: –0.220 and ${E}_{{{{{{\rm{T}}}}}}}^{{{{{{\rm{N}}}}}}}$: 0.767). 1,1,2,2-tetrafluoroethyl-2,2,3,3-tetrafluoropropyl ether (TTE) was projected onto a *β* vs. ${E}_{{{{{{\rm{T}}}}}}}^{{{{{{\rm{N}}}}}}}$ 2D plot (Supplementary Fig. 9d). Through UV-VIS measurements of each dye in the TTE solvent (Supplementary Fig. 9a), the calculated *β* was found to follow the trend both in the average CE and the (CIP + AGG)/SSIP ratio derived from Raman spectroscopy (Supplementary Fig. 9b, c).

After evaluating both the model solvents of our choice and the conventional FNSCs, we discovered the most suitable physicochemical properties describing the ideal non-solvating cosolvents. We also establish the correlation of lithium metal negative electrode performance with *β* and ${E}_{{{{{{\rm{T}}}}}}}^{{{{{{\rm{N}}}}}}}$, namely a low *β* (< 0.2) and a medium-high ${E}_{{{{{{\rm{T}}}}}}}^{{{{{{\rm{N}}}}}}}$ (> 0.11). For the non-fluorinated solvents to have such properties, there should be resonance structures involving the oxygen atom to endow the solvents with non-solvating and miscible characteristics. Based on our correlation results and design rules, we identified three candidate NFNSCs: AN, ethoxybenzene, and furan.

**Fig. 2:**
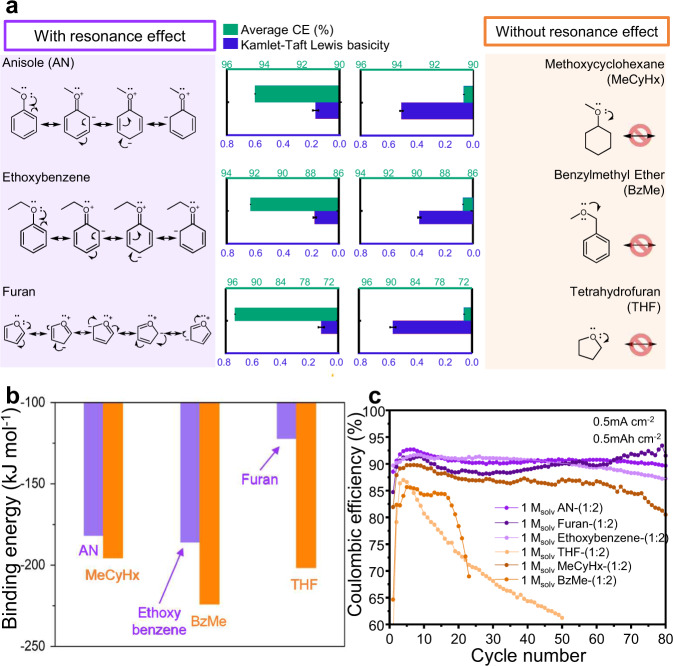
Revealing the origin of low lithium ion solvation ability of NFNSCs. **a** Resonance structures of organic compounds (with and without resonance effect which have purple and orange background, respectively) and the corresponding average CE and β values. **b** Binding energies of a lithium ion with the oxygen atom of each compound calculated in the gas phase (vacuum model). **c** Cycling performance of Li|Cu coin cells with different electrolytes at 0.5 mA cm^−2^ to 0.5 mAh cm^−2^. Error bars shown here are 95% CI.

A comparison with the non-resonant analogues, namely methoxycyclohexane (MeCyHx), benzylmethyl ether (BzMe), and tetrahydrofuran (THF), reveals that molecules with resonance structures involving lone pair electrons on the oxygen atom (With resonance effect in Fig. 2a) display lower *β* values, higher average CEs, and longer capacity retention compared to those with limited or no resonance structures (Without resonance effect in Fig. 2a) (Fig. 2a, c).

DFT calculations also suggested that the resonance can lower the binding energy of a lithium ion (Fig. 2b, Table S1). From the calculation, the binding energies of the lithium ion to the oxygen atom of AN, ethoxybenzene, and furan are significantly lower than those of analogous molecules with similar structures but different resonance capability. The same results were obtained under the implicit solvent model that contains EC: DEC = 1:1 (v/v) as a medium (Supplementary Fig. 10). Additional calculations were performed for several molecules with phenyl rings and oxygen atoms (Supplementary Figs. 11, 12). Comparing the oxygen atoms connected directly to the phenyl ring and those blocked by the methylene group, the former atoms exhibited lower binding energies, which once again confirms our hypothesis (Supplementary Note 5).

### Effect of AN on the electrochemical performance of electrolytes

We evaluated the electrochemical performance of 1 M_solv_ LiTFSI EC/DEC, 5 M_solv_ LiTFSI EC/DEC, and 1 M_solv_ LiTFSI EC/DEC:AN (1:4 v/v) electrolytes, which will be abbreviated as 1 M_solv_ EC/DEC, 5 M_solv_-HCE, and 1 M_solv_ AN-(1:4), respectively. The 1:4 (v/v) ratio of EC/DEC to AN was selected to maintain the local concentration of LiTFSI in EC/DEC at 5 M_solv_ and to investigate the dilution effect of AN in the LHCE.

**Fig. 3:**
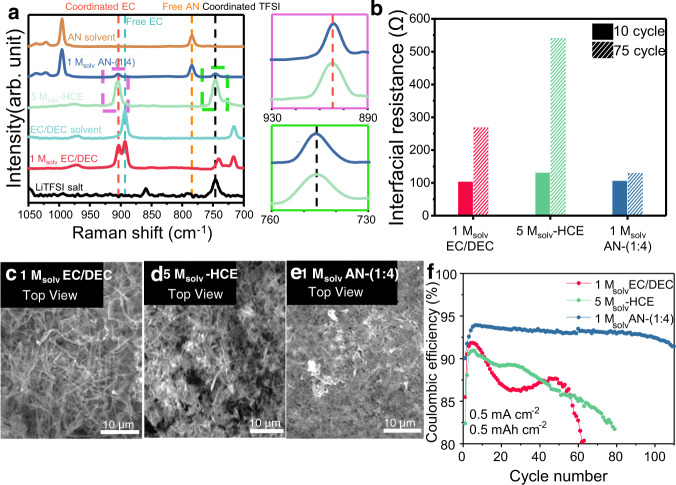
Comparing NFNSC-containing LHCE with conventional electrolyte and HCE. **a** Raman spectra of electrolytes and solvents containing LiTFSI, EC/DEC, and AN. **b** Interfacial resistance of different electrolytes at the 10th and 75th cycles obtained by fitting the EIS data. **c**–**e** Top-down SEM images of lithium deposition in (**c**) 1 M_solv_ EC/DEC, (**d**) 5 M_solv_-HCE, and (**e**) 1 M_solv_ AN-(1:4). **f** Cycling performance of Li|Cu coin cells using different electrolytes at 0.5 mA cm^−2^ to 0.5 mAh cm^−2^.

The Raman peak shift of TFSI^−^ for 1 M_solv_ AN-(1:4) was the same as that for 5 M_solv_-HCE and LiTFSI salts, confirming their identical solvation structures (Fig. 3a). In addition, the EC peaks of AN-(1:4) behaved like that of 5 M_solv_-HCE. Further deconvolution of the TFSI^−^ peak indicated that the ratio of CIP + AGG to SSIP increased as the ratio of AN increased (Supplementary Fig. 13). This LHCE solvation structure promotes the anion-derived SEI layer that was observed from the X-ray photoelectron spectroscopy (XPS) depth profile of the atomic ratios. Specifically, F and S, which comprise the inorganic SEI layer, were richer for 1 M_solv_ AN-(1:4) and 5 M_solv_-HCE compared to that for 1 M_solv_ EC/DEC (Figs. S14, 15).

Electrochemical performance of the Li|Cu cell with 1 M_solv_ AN-(1:4) demonstrated significantly improved cycle life and CE, even at high current densities and capacities (Fig. 3f, Supplementary Figs. 16, 17). The superior performance of the AN-containing electrolyte compared to 5 M_solv_-HCE and 1 M_solv_ EC/DEC can be attributed to the additional advantages of using non-solvating cosolvents. The solvation structure of the 1 M_solv_ AN-(1:4) electrolyte resembles that of 5 M_solv_-HCE, while it produces a more compact SEI layer and minimises electrolyte decomposition. These characteristics were characterised using scanning electron microscopy (SEM) and electrochemical impedance spectroscopy (EIS).

The morphology of lithium deposited in 1 M_solv_ AN-(1:4) was examined using SEM. This morphology was significantly more compact compared to other tested electrolytes (Fig. 3c–e, Supplementary Fig. 18). Furthermore, the cross-sectional SEM images indicated that 1 M_solv_ AN-(1:4) produced both the thinnest lithium metal layer and the smoothest surface (Supplementary Fig. 19). According to EIS analysis, the increment in interfacial resistance (the sum of charge transfer resistance and SEI layer resistance) from the 10th to the 75th cycle was the smallest in 1 M_solv_ AN-(1:4) (Fig. 3b, Supplementary Figs. 20, 21)^48^. This is further supported by the Li 1*s* XPS depth profile of the charged samples, where the lithium metal peak for 1 M_solv_ AN-(1:4) appeared closer to the surface, and the on-line electrochemical mass spectrometry (OEMS) data also showed less formation of CO_2_ and C_2_H_4_ gases in 1 M_solv_ AN-(1:4) (Supplementary Figs. 22, 23).

### Assessing the practicality of NFNSC for the lithium metal negative electrode

**Fig. 4:**
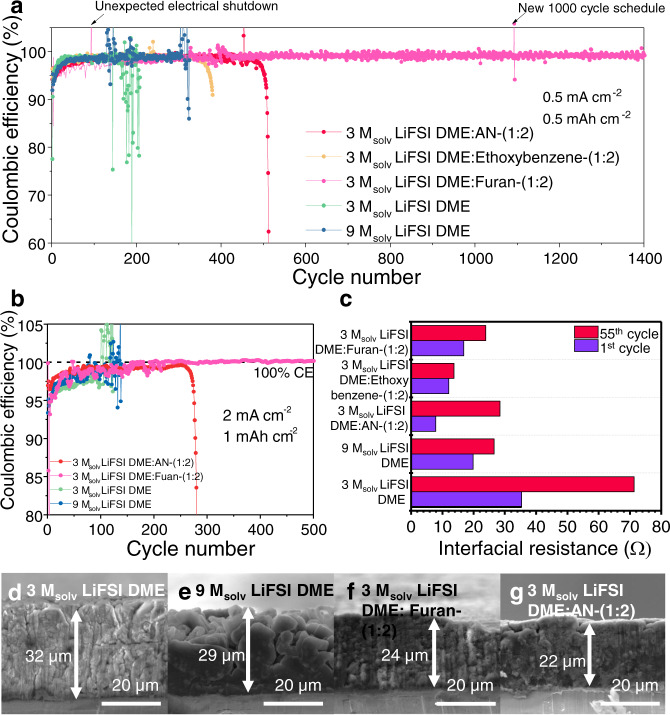
Cycling performance of Li|Cu coin cells with different electrolytes. **a**–**b** Cycling performance at (**a**) 0.5 mA cm^−2^ to 0.5 mAh cm^−2^ and (**b**) 2 mA cm^−2^ to 1 mAh cm^−2^. **c** Interfacial resistance of each electrolyte after the 1st and 55th cycles obtained from EIS analysis. **d**–**g** Cross-sectional SEM images of lithium metals deposited in (**d**) 3 M_solv_ LiFSI DME, (**e**) 9 M_solv_ LiFSI DME, (**f**) 3 M_solv_ LiFSI DME:Furan-(1:2), and (**g**) 3 M_solv_ LiFSI DME:AN-(1:2). The depth of the lithium metal layer was 32, 29, 24, and 22 μm, respectively.

Next, we further optimised salts and solvents in the electrolyte to improve the cell performance. After varying the salt concentration and volume ratio of the DME solvent to the cosolvent, both the 3 M_solv_ LiFSI DME:AN-(1:2) and 3 M_solv_ LiFSI DME:Furan-(1:2) systems showed the highest performance among the control groups (98.5% CE for 500 cycles and 99.0% CE for 1400 cycles) (Fig. 4a and Supplementary Figs. 24, 25). The solvent-to-cosolvent ratio was further applied to ethoxybenzene for a fair comparison among the NFNSC-containing electrolytes (Fig. 4a). The furan-containing electrolyte (3 M_solv_ LiFSI DME:Furan-(1:2)) attained an average CE of 99.0% after 1400 cycles, which is higher than those of 3 M_solv_ LiFSI DME and 9 M_solv_ LiFSI DME electrolytes. The electrolyte performance was also evaluated using the areal capacity *vs*. voltage profile and the overpotential profiles (Supplementary Figs. 26–28). Li|Cu cells containing 3 M_solv_ LiFSI DME:Furan-(1:2) performed the best even when the current density and capacity were increased to 2 mA cm^−2^ and 1 mAh cm^−2^, respectively (99.4% CE, 500 cycles) (Fig. 4b).

Full cell cycling tests were performed with lithium iron phosphate (LFP) as the positive electrode and electrodeposited thin lithium metal foil as the negative electrode. Different sets of experiments confirmed that the lithium metal had the areal capacity of ~3.49 mAh cm^−2^. Rate test with an LFP mass loading of ~11 mg cm^−2^ and thick lithium metal (300 μm) showed that at a current of 2 C, 3 M_solv_ LiFSI DME:AN-(1:2) and 3 M_solv_ LiFSI DME:Ethoxybenzene-(1:2) retained 79.5% and 68.7% of their respective capacity at 0.1 C (Supplementary Fig. 30a). These two electrolytes clearly have higher cyclability compared to 3 M_solv_ LiFSI DME and 1 M_solv_ LiPF_6_ EC:DMC-(1:1) (Supplementary Fig. 30b). At the 300^th^ cycle, they retained 93.7% and 71.4% of their respective initial capacity, whereas 3 M_solv_ LiFSI DME retained only 63.2%.

For furan-containing electrolytes, the optimal salt concentration for full cell is found lower, and the concentrations of 1 M_solv_ or 2 M_solv_ demonstrated the highest capacity retention among tested NFNSCs in Li|LFP full cells (94.8% and 86.2% retention of the respective initial capacity at the 300^th^ cycle, Supplementary Fig. 30).

At a higher LFP mass loading of 21 mg cm^−2^ with an N/P ratio of 1.6, the cells also showed 86.8% and 83.4% capacity retention at the 125^th^ cycle for 3 M_solv_ LiFSI DME:AN-(1:2) and 3 M_solv_ LiFSI DME:Ethoxybenzene-(1:2), respectively. In contrast, 1 M_solv_ LiPF_6_ EC:DMC-(1:1) failed directly after 20 cycles, and 3 M_solv_ LiFSI DME retained 78.9% of its initial capacity at the 120th cycle (Supplementary Fig. 31a). When using 1 M_solv_ and 2 M_solv_ furan electrolytes, the NFNSC-containing electrolytes showed higher capacity retention (Supplementary Fig. 31b). At the 125^th^ cycle, 1 M_solv_ LiFSI DME:Furan-(1:2) and 2 M_solv_ LiFSI DME:Furan-(1:2) showed 94.6% and 94.0% retention of their initial capacity, respectively.

The ionic conductivity significantly increased (by more than one order of magnitude) after adding the NFNSCs to 9 M_solv_ LiFSI DME HCE compared to that of 9 M_solv_ LiFSI DME (Supplementary Fig. 32)^15^. Even the ionic conductivity of 3 M_solv_ LiFSI DME:Furan-(1:2) is higher than that of 3 M_solv_ LiFSI DME. The interfacial resistance of NFNSC-containing electrolytes was compared with that of 3 M_solv_ LiFSI DME using EIS analysis. The electrolytes containing cosolvents showed smaller resistances in the 1st and 55th deposition cycle as well as a smaller resistance increase, confirming that the use of cosolvents resulted in a better SEI, which lowers the resistance (Fig. 4c, Supplementary Fig. 33)^48^.

Cross-sectional SEM images demonstrated that the lithium metals deposited in both 3 M_solv_ LiFSI DME:AN-(1:2) and 3 M_solv_ LiFSI DME:Furan-(1:2) were more compact and thinner than those deposited in 3 M_solv_ and 9 M_solv_ LiFSI DME (Fig. 4d–g, Supplementary Fig. 34). The ethoxybenzene-based electrolyte (3 M_solv_ LiFSI DME:Ethoxybenzene-(1:2)) produced a relatively thicker initial deposition, and this may be responsible for the lowest cyclability among NFNSC-containing electrolytes (Fig. 4a, Supplementary Fig. 35).

### Quantification of cosolvents in electrolytes

**Fig. 5:**
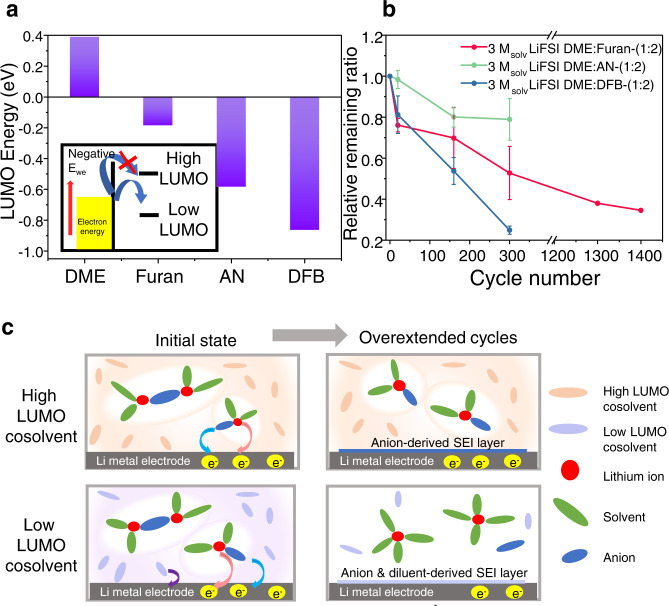
Effects of LUMO energy on cosolvent stability. **a** Calculated LUMO energy of different solvents (furan, DME, AN, and DFB) in mixed EC/DEC solvent (1:1 v/v). **b** Relative remaining ratio of furan, AN, and DFB after cycling the corresponding Li|Cu cells at 0.5 mA cm^−2^ to 0.5 mAh cm^−2^ for the designated number of cycles. **c** Schematic illustration of solvation structures in electrolytes containing cosolvents with high and low LUMO levels. Error bars shown here are 95% CI.

Electrolytes containing AN and furan demonstrated longer cycling than the reported cyclability of electrolytes added with DFB (350 cycles) and FB (500 cycles), which also contain fluorine and induce LiF formation on the lithium metal negative electrode^14,15^. In particular, when 3 M_solv_ DME:DFB-(1:2) was cycled using our electrochemical protocol, it failed earlier than 3 M_solv_ LiFSI DME:Furan-(1:2) (Supplementary Fig. 36). Despite the absence of beneficial fluorine atoms in the NFNSCs, we attribute the long cyclabilities of the NFNSC-containing electrolytes to the higher energy of their lowest unoccupied molecular orbital (LUMO). The LUMO level of a solvent is a dominant factor that determines the performance of an electrolyte, especially after extended cycling. A high LUMO level of the solvent guarantees its high stability under reductive condition^49,50^. Our quantum chemical calculations indicate that the LUMO levels of AN and furan are −0.584 and −0.186 eV, respectively; while that of DFB is substantially lower at −0.865 eV (Fig. 5a, Supplementary Table 2). The addition of electronegative fluorine to the molecule lowers the LUMO level, which aggravates the reduction reaction and facilitates cosolvent decomposition at the lithium metal negative electrode interphases^11,14,15^. Such facile cosolvent decomposition causes not only electrolyte but also a deviation of the overall composition from the initially designed electrolyte during cycling^22^ (Fig. 5c). We found that a decrease in the relative composition of cosolvents can decrease the CE after extended cycling and induce battery failure.

Linear sweep voltammetry (LSV) curves of 1 M_solv_ DFB-(1:2) during the initial charging exhibited additional reduction peaks at a higher voltage (Supplementary Fig. 37), which suggests a lower cathodic stability caused by a low LUMO. In addition, ^1^H NMR spectra were collected for electrolytes extracted from the Li|Cu cells after cycling, in order to quantify the concentration of the remaining cosolvents (refer to Supplementary Note 6 for the specific method). After 300 cycles, the quantities of remaining furan and AN were higher than those of DFB (Fig. 5b). Even after 1400 cycles, approximately 35% of furan remained, which is higher than the remaining ratios of DFB after the 300^th^ cycle. We did not detect any noticeable degradation of DME because of its higher LUMO level compared to that of other cosolvents in this study (Supplementary Fig. 41). This also suggests that the degradation of cosolvents is responsible for poor cycle retention.

**Fig. 6:**
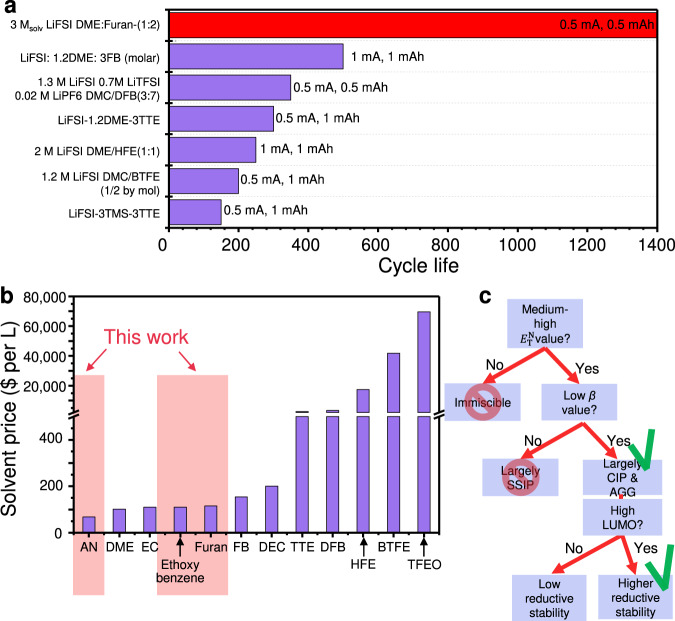
Comparison of reported cosolvents and proposed strategy for choosing NFNSCs. **a** Comparison of Li|Cu cell performance with various cosolvents reported in previous studies. The areal current density and areal capacity values are indicated. **b** Comparison of the prices of (co)solvents commonly utilised in the electrolyte of lithium metal negative electrode battery system. **c** A flowchart for choosing an appropriate NFNSC.

To the best of our knowledge, no previous conventional studies relying on expensive fluorinated cosolvents successfully overcame the cycle capability of lithium metal negative electrode for longer than 500 cycles with a CE value exceeding 99.0%^8,9,14,15,20,51^. Therefore, our design rule of the cosolvent opens a route for developing lithium metal negative electrode batteries with an exceptionally long cycle life (Fig. 6a). For a more objective comparison, we calculated the reversible accumulated capacity according to the information obtained in the literature using the following Eq. (1):

1 $$\begin{array}{c}{{{{{\rm{Reversible}}}}}}\,{{{{{\rm{accumulated}}}}}}\,{{{{{\rm{capacity}}}}}}\left({{{{{\rm{mAh}}}}}}\;{{{{{{\rm{cm}}}}}}}^{-2}\right)\\={{{{{\rm{Cycle}}}}}}\;{{{{{\rm{life}}}}}}\times {{{{{\rm{Areal}}}}}}\,{{{{{\rm{capacity}}}}}}\,\left({{{{{\rm{mAh}}}}}}\,{{{{{{\rm{cm}}}}}}}^{-2}\right)\times {{{{{\rm{Average}}}}}}\,{{{{{\rm{CE}}}}}}\left(\%\right)\end{array}$$

The 3 M_solv_ LiFSI DME:Furan-(1:2) electrolyte system was found out to produce the highest reversible accumulated capacity among various electrolytes that adopt cosolvents (Supplementary Fig. 43).

Along with the CE and cyclability, the cost of electrolytes is a major criterion for assessing their practicality. The prices of solvents investigated in this work are compared with other cosolvents in Fig. 6b and Supplementary Table 3. Many cosolvents reported to date are considerably more expensive than the electrolyte solvents used in practical lithium ion battery such as EC, DMC or DEC. The high price of cosolvents should increase the cost of battery production. Common fluorine-free organic compounds such as AN, furan, and ethoxybenzene are much cheaper and actually comparable to common solvents such as EC, DEC, and DME.

Based on the above information, we propose a flowchart for selecting an appropriate non-solvating cosolvent for use with high-performance lithium metal negative electrodes. The cosolvent should have a medium-to-high ${E}_{{{{{{\rm{T}}}}}}}^{{{{{{\rm{N}}}}}}}$ (>0.11) to be miscible with polar electrolytes, a low *β* value (<0.2) to induce an anion-coordinated solvation structure, and a high LUMO level (>−0.6 eV according to our calculation) to remain stable under highly reductive conditions (Fig. 6c).

In this study, we proposed critical parameters for choosing the optimal non-solvating cosolvents for LHCEs and correlated their effects with the electrochemical performance of various electrolytes. These insights enabled us to replace the conventional FNSC with NFNSCs that show superior cathodic stability, as confirmed by the long cyclability of the 3 M_solv_ LiFSI DME:Furan-(1:2) electrolyte. This work represents a significant advancement in designing non-solvating cosolvents by providing new ways to tune the solvation ability of a molecule without sacrificing its reductive stability. In addition, the design rule proposed here is useful for determining the solvation ability and predicting the performance of candidate solvents in lithium metal batteries. Overall, these results would advance the design of electrolytes for high-performance lithium metal negative electrodes.

## Methods

### Materials and electrolyte preparation

LiTFSI (99.95%, trace metal basis), ethoxybenzene (99%), fluorobenzene (99%), benzylmethyl ether (98%), dibenzyl ether (>98%), diphenyl ether (>99%), 1,2-dimethoxyethane (99.5%, anhydrous, inhibitor free), ethylene carbonate (EC, 99%, anhydrous), diethyl carbonate (>99%, anhydrous), dibutyl ether (99.30%, anhydrous), 1,3-dioxolane (99.80%, anhydrous, containing ~75 ppm BHT as inhibitor), furan (>99%), and 4-nitroaniline (>99%) were purchased from Sigma-Aldrich. LiFSI (>98%), 1,2-difluorobenzene (>98%), methoxycyclohexane (>98%), 1,1,2,2-tetrafluoroethyl-2,2,3,3-tetrafluoropropyl ether (>95%), and diisopropyl ether (>99%) were purchased from Tokyo Chemical Industry Co. Ltd. AN (99%), methyl tert-butyl ether (HPLC grade, anhydrous), tetrahydrofuran (99.8 + %, anhydrous), 1,4-dioxane (anhydrous, 99.8%, stabilised with 1–3 ppm BHT) was purchased from Alfa Aesar. Deuterated dimethyl sulfoxide (DMSO-d^6^, >99.8%) as NMR solvent was purchased from Deutero. *N*,*N*-Diethyl-4-nitroaniline (98%) was purchased from Combi-Blocks. All solvents except EC were stored with 4 Å molecular sieve (Alfa Aesar) for at least two days, and all electrolytes were prepared in an Ar-filled glove box. Because the volume ratio of EC/DEC was fixed at 1:1 for all experiments, we did not explicitly indicate this ratio in the main article. In this article, the concentration of the electrolyte (M_solv_) is defined as the mole of salt dissolved in a litre of the solvent^39–41^.

### Calculating the solvatochromic *β* parameter

The *β* parameter was calculated based on the method proposed by Kamlet *et al*.^42^ The dye solution of 4-nitroaniline (NA, Tokyo Chemical Industry) and N,N-diethyl-4-nitroaniline (DA, Combi-Blocks) were put in the cosolvent of which we want to calculated the *β* parameter making the final concentration of each dye to 1.0 × 10^−4^ M. The absorption spectra were measured using a Shimadzu UV-2600 spectrophotometre in the spectral range of 300–500 nm with a resolution of 0.1 nm.

Then, the maximum absorption wavelength of each dye solution (*λ*_max_(NA) and *λ*_max_(DA)) was used in the following ways to derive *β* parameter of each cosolvent.

1. *λ*_max_(NA) and *λ*_max_(DA) each converted from nm scale to kK scale (1 kK = 1000 cm^−1^).
2. Converted *λ*_max_(DA) was set to new values by multiplying 1.035 and adding 2.64.
3. By subtracting the converted *λ*_max_(NA) from the obtained value in 2nd step, and then dividing by 2.80, we can obtain the *β* parameter of each cosolvent.

The scientific significance of each step is mentioned in Physicochemical properties of NFNSCs and their design strategy, Results section and Supplementary Note 2.

### Calculating the solvatochromic ${E}_{{{{{{\rm{T}}}}}}}^{{{{{{\rm{N}}}}}}}$ parameter

The ${E}_{{{{{{\rm{T}}}}}}}^{{{{{{\rm{N}}}}}}}$ parameter was calculated based on following method^46^.

1. A mother solution of 2,6-diphenyl-4-(2,4,6-triphenyl-1-pyridino) phenolate (Reichardt’s dye) in TTE was prepared at a concentration of 1 × 10^–2^ M.
2. The mother solution was diluted to 1 × 10^–4^ M with TTE, and the absorption spectrum was measured using a Shimadzu UV-2600 spectrophotometre. The spectral range was 400–700 nm, and the resolution was 0.1 nm.
3. The *λ*_max_ value was recorded from the recorded spectrum (shown in Supplementary Fig. 9a), and the *E*_T_(30) value was calculated using the relation *E*_T_(30) = 28591/*λ*_max_.
4. The *E*_T_(30) values were normalised using the values of water and tetramethylsilane (TMS), and finally the ${E}_{{{{{{\rm{T}}}}}}}^{{{{{{\rm{N}}}}}}}$ value was obtained.

2 $${E}_{{{{{{\rm{T}}}}}}}^{{{{{{\rm{N}}}}}}}=\frac{\big(E_{{{{{{\rm{T}}}}}}}(30,{{{{{\rm{sovlent}}}}}})-(E_{{{{{{\rm{T}}}}}}}(30,{{{{{\rm{TMS}}}}}}))}{\big(E_{{{{{{\rm{T}}}}}}}(30,{{{{{\rm{water}}}}}})-(E_{{{{{{\rm{T}}}}}}}(30,{{{{{\rm{TMS}}}}}}))}$$

### Electrochemical testing

The 2032-type coin cells were assembled in an Ar-filled glove box. A Celgard 2320 microporous membrane separator and 80 μL of electrolyte were used in each cell. All cycling experiments were performed at 25 °C using a WBCS3000L battery cycler (WonATech). The Li|Cu cells were rested for 3 h at the open-circuit voltage (OCV) and then cycled at the predetermined areal current and capacity. The cut-off voltage for each cycle was set to 1 V (vs. Li/Li^+^). The average CE was measured using the modified Aurbach method while the current density was fixed at 0.5 mA cm^−2^. The initial pre-cycle (deposition and stripping) was performed to 5 mAh cm^−2^, followed by the deposition of 5 mAh cm^−2^ Li (*Q*_R_, amount of charge). The final stripping (cut-off voltage: 1 V) was performed (*Q*_S_, amount of charge) after 10 deposition and stripping cycles to 1 mAh cm^−2^. Therefore, the average CE is calculated as follows:

3 $${{{{{\rm{Average}}}}}}\,{{{{{\rm{CE}}}}}}(\%)=\frac{({Q}_{{{{{{\rm{s}}}}}}}+1\times 10){{{{{\rm{mAh}}}}}}\,{{{{{{\rm{cm}}}}}}}^{-2}}{({Q}_{{{{{{\rm{R}}}}}}}+1\times 10){{{{{\rm{mAh}}}}}}\,{{{{{{\rm{cm}}}}}}}^{-2}}\times 100\left(\%\right)$$

EIS and cyclic voltammetry (CV) were performed using a VSP-300 (BioLogic) on Li|Cu asymmetric cells. Both experiments were carried out at room temperature after resting the cell at the OCV for 30 s. EIS measurements were conducted after the discharge cycle, in the frequency range from 100 kHz to 0.1 Hz with a 10 mV amplitude.

### Characterisation

After disassembling the cells in an Ar-filled glove box, the electrodes were washed with DEC or DME and dried under vacuum. SEM (MIRA3 XMH, TESCAN) was used for morphological characterisation. The loaded SEM samples were placed directly into the chamber to minimise contact with air. The accelerating voltage was set to 15 kV.

The chemical compositions and bonding characteristics of the SEI layers were analysed using XPS (Thermo VG Scientific (Sigma Probe, Mg Kα source)). All peaks were fitted according to the reference peak of C–C bond at 284.8 eV. A step size of 1.0 eV was used for the survey scan, and a step size of 0.1 eV was used for high-resolution scans for all elements.

The coordination structures of various electrolytes were studied by Raman spectroscopy using an inVia Raman microscope (Renishaw) with an excitation laser of 514 nm. Peak deconvolution of the obtained spectra was performed using a Gaussian function. At least three spectra were obtained on different days for each electrolyte.

To quantify the cosolvents and salt, Li|Cu coin cells containing 40 μL of electrolytes were subjected to a specific number of cycles at 0.5 mA cm^−2^ to 0.5 mAh cm^−2^. All disassembled parts of the coin cells were placed in a polytetrafluoroethylene vial with 1 mL of DMSO-d_6_. EC (20 μL) was used as an internal standard for ^1^H NMR. After shaking the vials for 5 min^52^, ^1^H NMR spectra were collected on a 500 MHz NMR system (Varian 500). Peak deconvolution was performed with the Mestrenova software using the Lorentzian fit method. This process was repeated at least thrice for each sample.

### Computational details

Quantum chemical calculations were performed with Gaussian 16 (Revision A.03) using density functional theory (DFT) at the B3LYP/6-31 G+(d,p) level of theory with Grimme dispersion (D3BJ) and ultrafine integration^53–56^. To account for the solvation effects, the calculations were performed in vacuum and with the IEFPCM implicit solvation model. As we set the volume ratio of EC:DEC to 1:1 in experiments, using the density value of EC (1.32 g cm^−3^) and DEC (0.98 g cm^−3^) and molecular weight of EC (88.06 g per mol) and DEC (118.13 g per mol) respectively, the molar ratio of EC:DEC in our experiments is 1.81:1. The dielectric constant of EC:DEC at 1.81:1 molar ratio was calculated as *ε*_(1.81:1 EC:DEC)_ = *ε*_EC_ *X*_EC_+*ε*_DEC_ *X*_DEC_ using the dielectric constants at 25 °C of EC (89.78^57^) and DEC (2.82^58^), where *X* signifies the molar fraction and *ε* signifies the dielectric constant. The binding energies and LUMO energy levels were determined from the obtained structures.

## Supplementary information

Supplementary Information

## Data Availability

Most data supporting the findings of this study are available from the main text of this article and its Supplementary Information. Raw datasets can be obtained from the corresponding author on reasonable request.
