# Supplementary material for: Non-fluorinated non-solvating cosolvent enabling superior performance of lithium metal negative electrode battery
Source: Nat Commun. 2022 Aug 4;13:4538. doi: 10.1038/s41467-022-32192-5 (PMC9352671; doi:10.1038/s41467-022-32192-5)
Supplement: Supplementary file 1 — Supplementary Information [file 41467_2022_32192_MOESM1_ESM.pdf]

Non-fluorinated non-solvating cosolvent enabling superior performance of lithium metal negative electrode battery

Junyeob Moon[1,†], Dong Ok Kim[1,2,†], Lieven Bekaert[3], Munsoo Song[1], Jinkyu Chung[1], Danwon Lee[1], Annick Hubin[3,*], Jongwoo Lim[1,4*]

[1] Department of Chemistry, College of Science, Seoul National University, Seoul, 08826, Republic of Korea

[2] Department of Chemistry and Biochemistry, Swarthmore College, Swarthmore, PA 19081, United States of America

[3] Vrije Universiteit Brussel, Research Group Electrochemical and Surface Engineering, Pleinlaan 2, 1050, Brussels, Belgium

[4] Molecular Science Research Institute (MSRI), Seoul National University, Seoul, 08826, Republic of Korea

[†] These authors contributed equally: Junyeob Moon, Dong Ok Kim

[*] Corresponding authors, e-mail: annick.hubin@vub.be, jwlim@snu.ac.kr

Supplementary Methods

Materials

Lithium iron phosphate (LFP) was purchased from MTI, super P was purchased from TIMCAL, N-methyl-2-pyrrolidone (99.5+%, anhydrous) was purchased from Acros Organics and polyvinylidene fluoride (PVDF) binder (7208) was purchased from Kureha.

Electrochemical Testing

Thin foil lithium metal was electrodeposited on copper foil using lithium metal as a counter electrode and LiFSI:DME:TTE (1:1.2:3 in molar ratio) as an electrolyte. To make a larger scale thin foil lithium metal, we used pouch cell configuration with 25 cm^2 copper foil. Resting the pouch cell for 12 hours, we applied 0.5 mA cm^{-2} for 8 hours and then the electrodeposited foil was taken out, rinsed thoroughly with DME solvent and then dried under vacuum to evaporate residual solvent. Through depleting electrodeposited thin foil lithium metal, the discharging capacity was found out to be 3.49 mAh cm^{-2}.

For the full cell, LFP (MTI) was used as a cathode material. First experiment, the LFP, Super P, and PVDF binder were set to a 8:1:1 weight ratio (mass loading ~11 mg cm^{-2}) and mixed with N-methyl-2-pyrrolidone. Second experiment, we set the LFP, Super P, and PVDF binder weight ratio to 93.5:4:2.5 (mass loading ~21 mg cm^{-2}) and mixed with N-methyl-2-pyrrolidone. An electrodeposited thin foil lithium (Li) metal (4 mAh cm^{-2}) was fabricated with Li|Cu pouch cell with pressure containing LiFSI:DME:TTE (1:1.2:3 in molar ratio) electrolyte[1]. The lithium metal was electrodeposited with 0.5 mA cm^{-2} for 8 hours. Then, we used 10 mm diameter LFP slurry and 12 mm diameter thin foil lithium metal. The LFP|Li coin cell was rested for 24 h before each

measurement. Then, for 80 wt% LFP experiment, 2 formation cycles were run at 0.1 C and then cycled at 0.5 C; for 93.5 wt% LFP experiment, 2 formation cycles were run at 0.05 C and then cycled at 0.2 C. The cyclic voltammetry (CV) scan rate was 1 mV s^{-1}. The cell was scanned from open-circuit voltage (OCV) to 0 V (vs Li/Li^+) and reversed back to 2 V (vs Li/Li^+) three times. Oxidative stability of NFNSC-containing electrolytes was checked with Al|Li coin cell LSV starting from OCV up to 5 V (vs. Li/Li^+) with scan rate of 1 mV s^{-1}.

Ionic conductivity was measured through EIS. With frequency range from 1 MHz to 1 Hz, we obtained Nyquist plot and fitted in order to obtain bulk resistance. The ionic conductivity of each electrolyte was obtained through the following equation, where σ is ionic conductivity (S cm^{-1}), A is area of electrode, l is the distance between two electrodes, and R is bulk resistance measured from EIS

$$\sigma = \frac{l}{RA} \quad (1)$$

Online electrochemical mass spectrometry (OEMS) Measurement

OEMS (Hiden Analytical) was used to detect the gas evolution in the battery during the cycle in real time. Calibrations were performed with various gases of different inlet pressures by constructing 3D contour plots to obtain quantifiable results. The gases of interest were quantified by relating m/z signals collected from the mass spectrometer to partial pressures of each gas in the carrier gas. The Cu|Li OEMS cells were assembled with a 38 mm Li foil with 16 holes (2 mm in diameter), 47 mm glass fibre separator, and 40 mm Cu foil. Exactly 1 ml of electrolyte was used for all cells to quantify the gas evolution induced by electrolyte decomposition.

Supplementary Notes

Supplementary Note 1. Candidates of solvation ability parameter

The DFT binding energy exhibited the lowest correlation with the CE of an electrolyte despite it being one of the most popular parameters in predicting the thermodynamics of solvents and salts. The correlation for the Gutmann donor number was −0.728 which is lower than that for the β value as mentioned in the main article (Supplementary Figure 1b).

Su *et al.* suggested relative solvating power—the ratio between the lithium coordination percentage of a solvent of interest and that of a reference solvent—to evaluate the ability of a solvent to solvate lithium ions[2]. However, this method requires the reference solvent to be the denominator, and therefore, it is limited to an electrolyte that comprises only one other solvent besides the reference solvent. Thus, an appropriate parameter that can be utilized for evaluating the lithium ion solvation ability of the solvents in the electrolyte system with three or more solvents is required. The electrolyte system utilized in this study comprises three solvents: EC, DEC and a co-solvent. Thus, this study adopted another parameter for the lithium ion solvation ability.

Supplementary Note 2. Calculating solvatochromic β parameter through UV-VIS spectrometer

The Lewis acid-base interaction is deterred and only van der Waals interaction is effective because of the two ethyl groups attached to the nitrogen atom in N,N-diethyl-4-nitroaniline (DA). Further, 4-nitroaniline (NA) realizes Lewis acid-base and its van der Waals interaction with its surrounding solvent (Supplementary Figure 3).

To deconvolute the effect of Lewis acid-base interaction to the candidate solvents, the van der Waals interaction should be subtracted. However, because of the structural difference between DA and NA, the portion of van der Waals interaction should be tuned. In order to tune the van der Waals interaction from two dyes same, we used the linear correlation obtained from the spectroscopic results of several nonpolar solvents

$$\upsilon(\text{NA, w/o-AB})_{\text{max, calculated}} = 1.035\times\upsilon(\text{DA, w/o-AB})_{\text{max, observed}} + 2.64\ \text{kK}\ (\text{kK: kiloKaiser, 1kK} = 1000\ \text{cm}^{-1})\quad(2)$$

because under the condition, both NA and DA only show the van der Waals interaction with surrounding solvents. Then, $\upsilon(\text{NA, w/o-AB})_{\text{max, calculated}}$ represent the van der Waals interaction of NA without Lewis acid-base interaction with surrounding solvents. The only difference between the experimentally obtained wavenumber of NA (i.e. $\upsilon(\text{NA, w/AB})_{\text{max, observed}}$) and the calculated value (i.e. $\upsilon(\text{NA, w/o-AB})_{\text{max, calculated}}$) is dependent on the Lewis acid-base interaction; this is equivalent to the lithium ion solvation ability of each solvent[3]. Finally, we can obtain β value after dividing the difference by 2.80. The raw data for UV-VIS measurements of each dye is shown in Supplementary Figure 3b and the process is summarized as a schematic in Supplementary Figure 3c.

Supplementary Note 3. Additional comments on the miscible behaviour of electrolytes

Various parameters for polarity were evaluated after establishing β as the lithium ion solvation ability parameter. They were plotted against β values to find the two-dimensional plot that best represents the miscibility behaviours of actual electrolytes observed; the E_T^N value was selected as the most appropriate polarity parameter as a result (Supplementary Figure 1c-d)[4].

Solvents that are not miscible with EC/DEC electrolyte system are DIE, DBE, and DPE. Without adding the salt, DPE shows miscible behaviour with EC/DEC whereas DEE, DIE, and DBE

show immiscible behaviour (Supplementary Figure 6). This can be attributed to the high E_T^N value of the EC/DEC mixture (polarity index of EC-E_T^N: 0.552, DEC-E_T^N: 0.182))[4]. Although the E_T^N value of DEC is low, the 1:1 ratio mixture of EC/DEC may have higher polarity as EC is added to the system[5]. Thus, it is plausible that DPE, which has the highest E_T^N value among four solvents (DPE, DEE, DIE, and DBE), is miscible with EC/DEC.

When adding the LiTFSI salt to the EC/DEC and solvent mixture, DEE becomes miscible while DPE becomes immiscible (Supplementary Figure 1a). This phenomenon may be attributed to the low β value of DPE. In addition, DIE and DBE retain their immiscible nature.

Supplementary Note 4. Deconvolution of Raman spectra of ether solvents

We obtain the Raman spectra of each electrolytes containing various solvents by utilizing our lab-made pouch cell configuration (Supplementary Figure 4a). Whether the β value accurately represents the actual solvation structure is examined by Raman spectroscopy and following $TFSI^-$ peak deconvolution (Supplementary Figure 4b-c). DEE and DOL did not fit exactly into this trend, which is because of their unique, physical properties to loosely coordinate with lithium ions despite their high β values [6,7].

Supplementary Note 5. Analysis on additional molecules analogous to AN

Molecules shown in Supplementary Figure 11 are used for this analysis. In Supplementary Figure 12a, lithium ion shows a lower binding energy to the O atom than to the phenyl group in the AN molecule. Further, the lithium ion shows a higher binding energy to the O atoms than to the phenyl groups in other molecules: BzMe, 2C-BzMe, and 3C-BzMe. This phenomenon is caused by the delocalisation of oxygen lone-pair electrons in AN. A similar trend is observed as

an increasing number of carbons are added to the terminal methyl group of AN (Supplementary Figure 12b). The reverse trend of propylphenyl ether may be attributed to the increased inductive effect of the alkyl chain. Finally, in the PMMB molecule, the lithium ion has lower binding energies to the O atom whose lone-pair electrons are delocalized compared to those of the O atom whose lone-pair electrons are not (Supplementary Figure 12c). It is unclear whether the strong binding of lithium ion in the blocked position can be attributed to its interaction with both the oxygen atom and the phenyl ring simultaneously. This is investigated by calculating the binding energy on PM2MB. In the PM2MB molecule, the binding energy of lithium ion to the blocked oxygen atom is not as strong as the energy calculated for the PMMB molecule (Supplementary Figure 12c). However, it is still stronger than the binding energy of the lithium ion to the delocalised oxygen atom, which confirms that the delocalisation plays a pivotal role in lowering binding energy between the lithium ion and the solvent.

Supplementary Note 6. Quantification in ^{1}H-NMR spectroscopy

The quantification of the electrolyte component via NMR spectroscopy entails possible error sources such as pipetting electrolytes in the coin cell, leakages during assembly, and evaporation of the solvent while preparing samples. Owing to the numerous sources of error, the absolute quantities of furan and AN varied considerably when samples were collected from disassembled coin cells (Supplementary Figure 39-40). Although the samples were collected from the uncycled cells, the values of the remaining AN were found to be smaller than of those obtained from pristine electrolytes (Supplementary Figure 38). The deviation decreases with a change in the parameter to the cosolvent (furan, AN, and DFB) to solvent (DME) ratio (Supplementary Figure 40). We determined that this ratio is appropriate for quantifying the cosolvents because

there was no noticeable degradation of DME as the cycles proceed (Supplementary Figure 41). Further, from the integrated coin cell, a smaller amount of electrolyte components was extracted compared to the initially added 40 μL of electrolyte because of the wetting of the Celgard 2320 separator. Therefore, samples extracted from the non-cycled coin cell were used as reference of full 40 μL electrolytes. NMR peaks used for the quantification of electrolyte components are indicated in the raw NMR spectra (Supplementary Figure 42).

Supplementary Figures

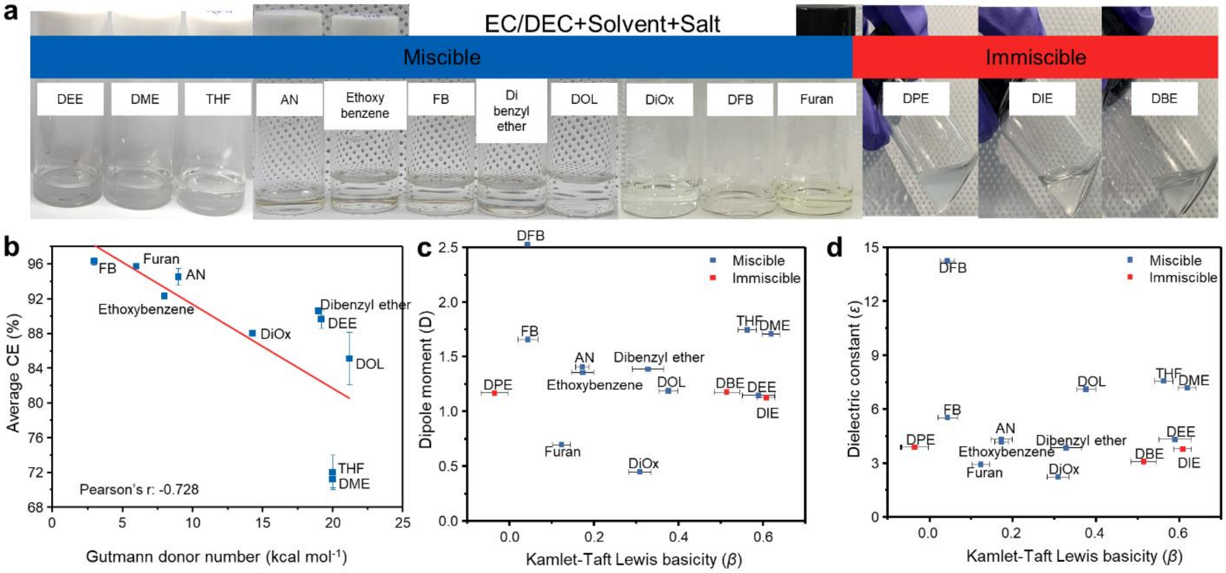

Supplementary Figure 1. **Finding optimal parameters representing lithium ion solvation ability and miscibility behaviour. a** Optical images of miscible electrolytes (1 M_{solv} LiTFSI EC/DEC:Solvent-(1:2)). Each denoted for its constituting solvent. Their full names and abbreviations are written on Supplementary Figure 2. **b** Correlation between the average CE and Gutmann donor number. **c** Kamlet-Taft Lewis basicity vs dipole moment two-dimensional plot of each solvent. **d** Kamlet-Taft Lewis basicity vs dielectric constant two-dimensional plot of each solvent. Error bars shown here are 95% confidence interval (CI).

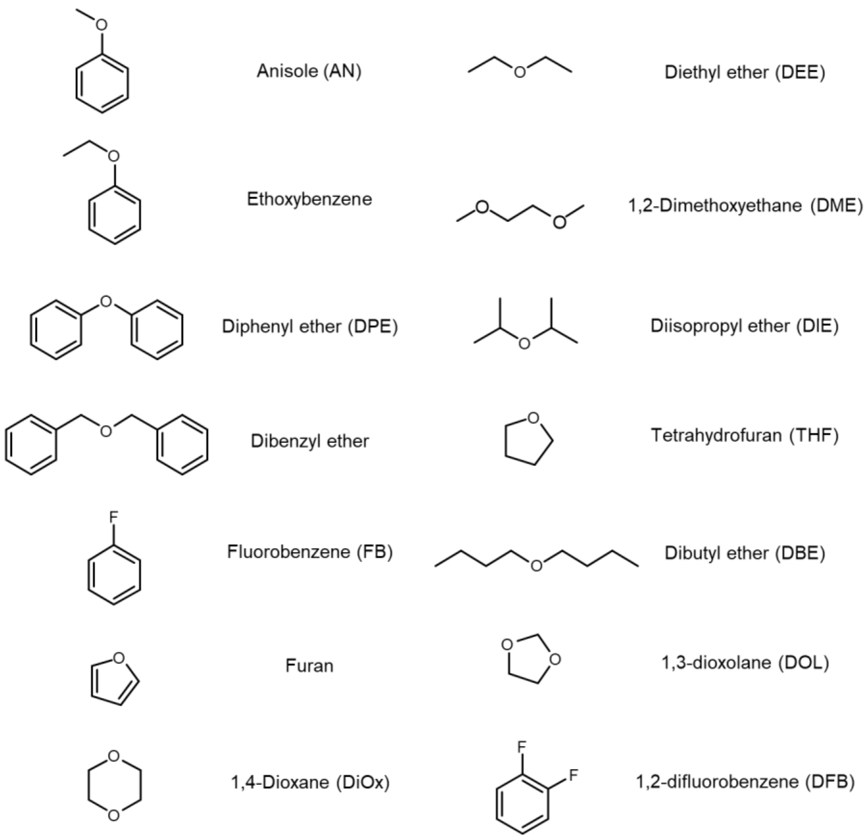

Supplementary Figure 2. **The structures of organic molecules studied in Fig. 1.** The upper-case letters in the parenthesis shows the abbreviation used in the main text and supplementary materials.

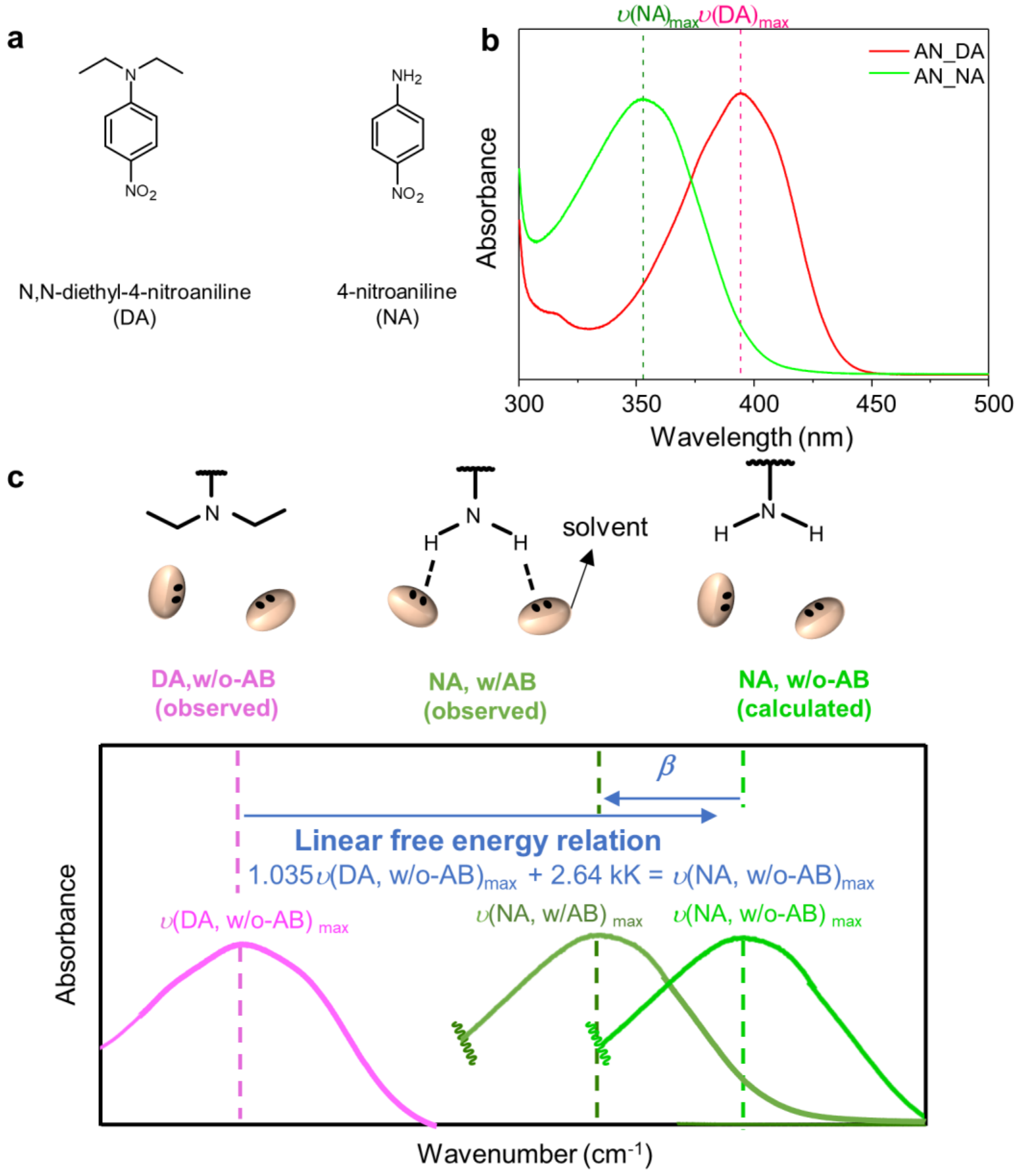

Supplementary Figure 3 Calculating Kamlet-Taft Lewis basicity (*β*). a Molecular structure of dyes used for calculating *β* value. **b** Representative UV-VIS spectrum of AN containing DA and NA dyes, respectively. **c** Schematic of how *β* was calculated through UV-VIS measurements. Here, w/o-AB stands for 'without Lewis acid-base interaction' and w/AB stands for 'with Lewis acid-base interaction'.

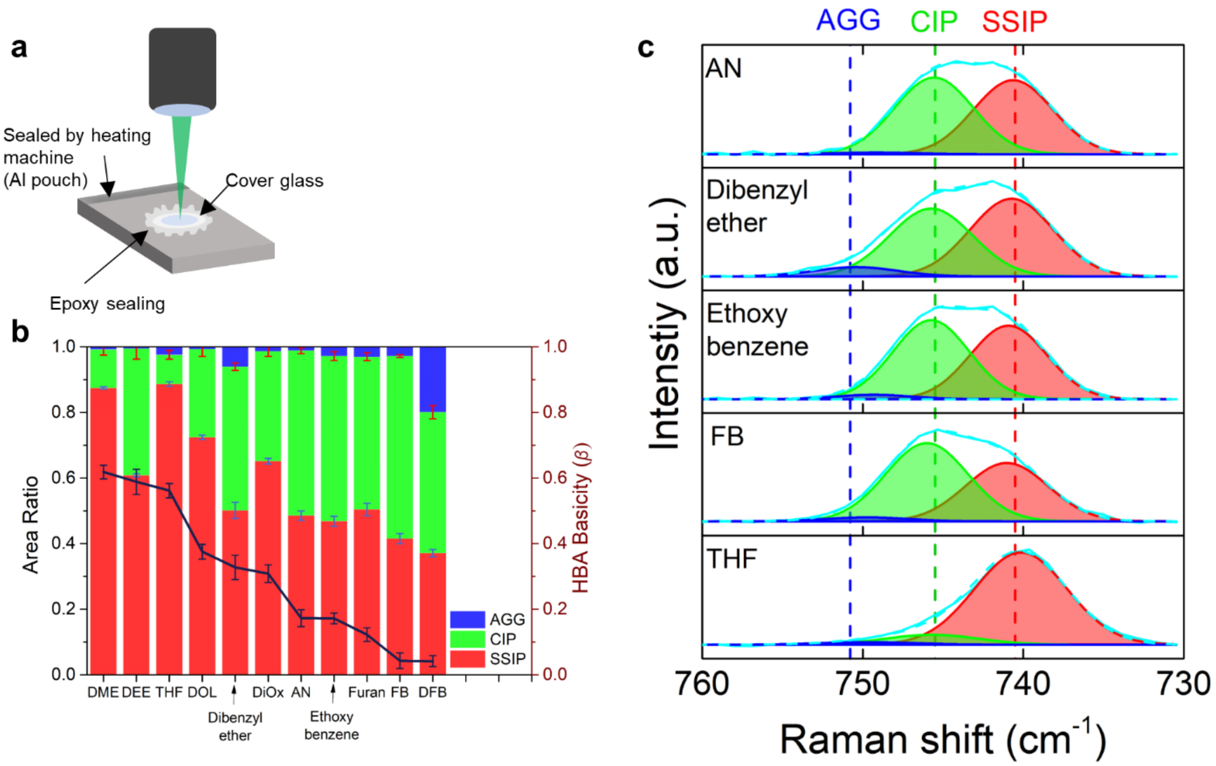

Supplementary Figure 4. Revealing lithium ion solvation structure of electrolytes through Raman spectroscopy. a Schematic illustration of a sample prepared for Raman spectroscopy. To avoid any air exposure of the samples, the electrolyte solution or salt was put in a pouch and sealed in a glove box. Before putting the solution in, a hole was made using a hole puncher on a pouch and was sealed with cover glass using epoxy glue. **b** Area ratio of Raman deconvoluted peaks of cosolvents-containing electrolytes (each denoted as their corresponding cosolvents' name) and their according Kamlet-Taft Lewis basicity. Refer to Supplementary Note 3 for more discussion. **c** Representative Raman peak deconvolution for $TFSI^-$ shift of electrolytes containing different cosolvents. Error bars shown here are 95% CI.

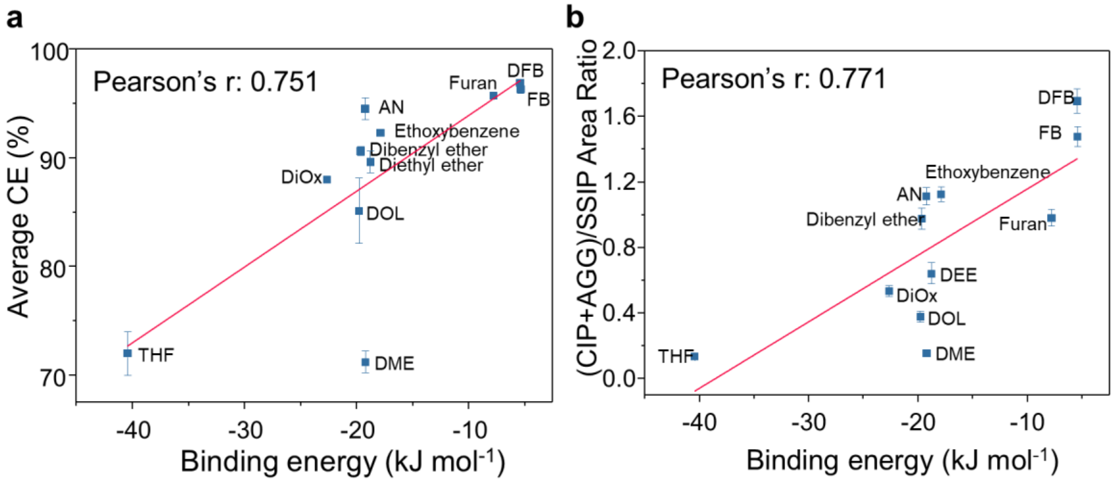

Supplementary Figure 5. Correlation of calculated binding energy under solvent model with various performance indexes. a Correlation between the average CE and the calculated binding energy (solvent condition, EC:DEC=1:1 v/v) **b** Correlation between the (CIP+AGG)/SSIP area ratio and the calculated binding energy (solvent condition). Error bars shown here are 95% CI.

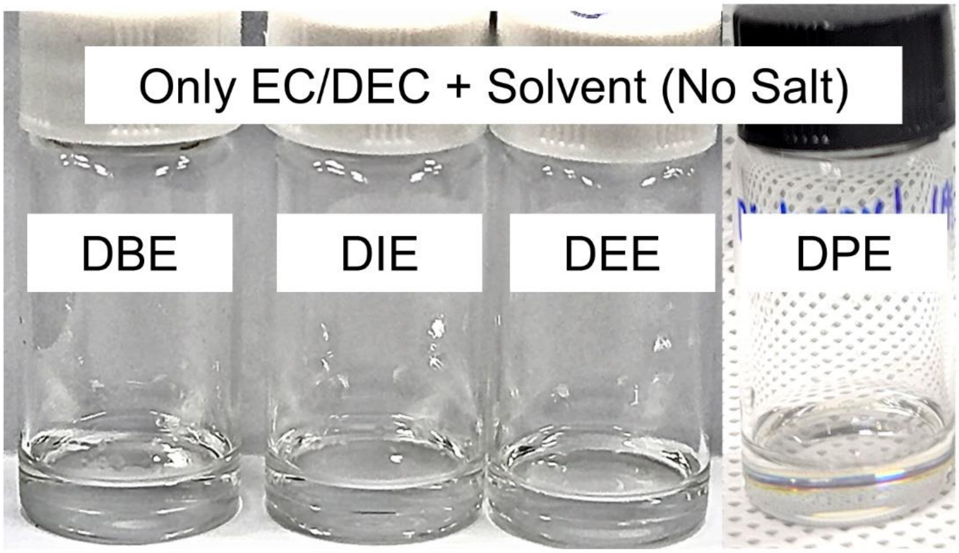

Supplementary Figure 6. Visual images of corresponding solvent mixture with EC/DEC (1:1 v/v). DBE, DIE and DEE are miscible with EC/DEC (1:1, v/v), but DPE is not miscible.

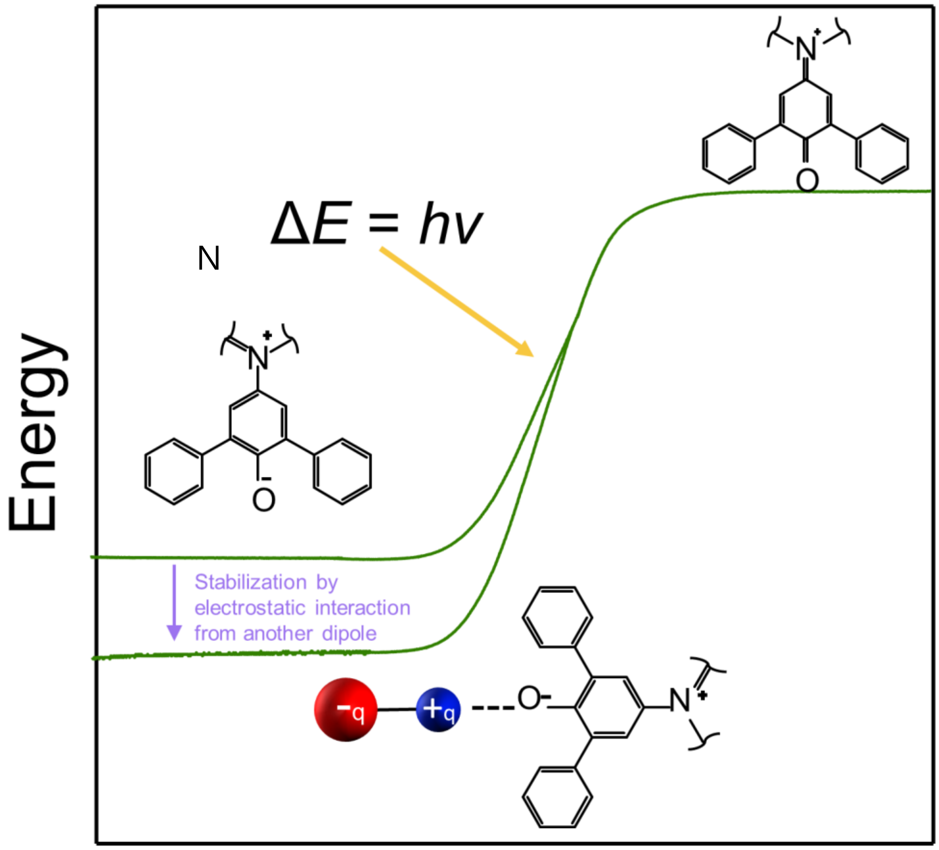

Supplementary Figure 7. The schematic of E_T^N (normalized electronic transition energy) measurement. Note that phenolate part mainly interacts with the solvent because of the substantial steric hindrance of benzene rings surrounding nitrogen atom.

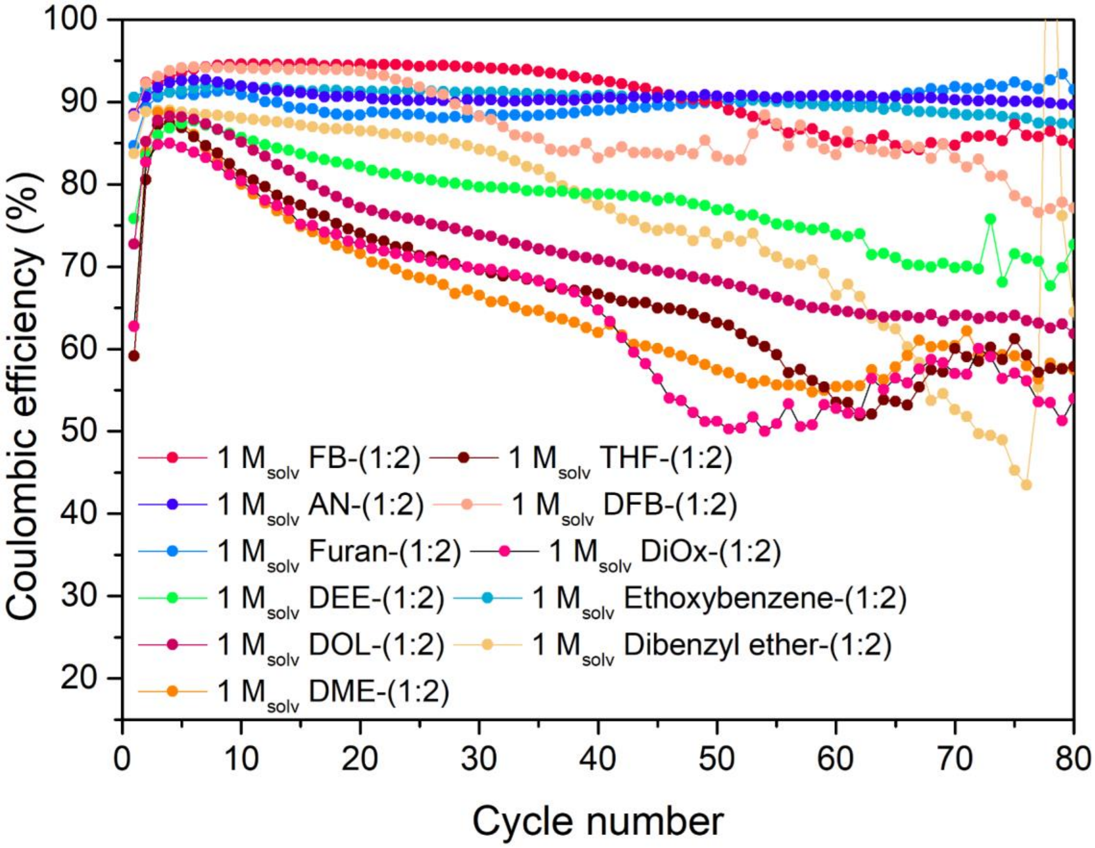

.

Supplementary Figure 8. Cycling performance of Li|Cu cells under various electrolytes. Li|Cu cells cycled at 0.5 mA cm^{-2} to 0.5 mAh cm^{-2}, containing cosolvents investigated in Fig. 1. As stated in main manuscript, we used 1 M$_{solv}$ LiTFSI EC:DEC:cosolvent (1:1:4 volume ratio) electrolytes.

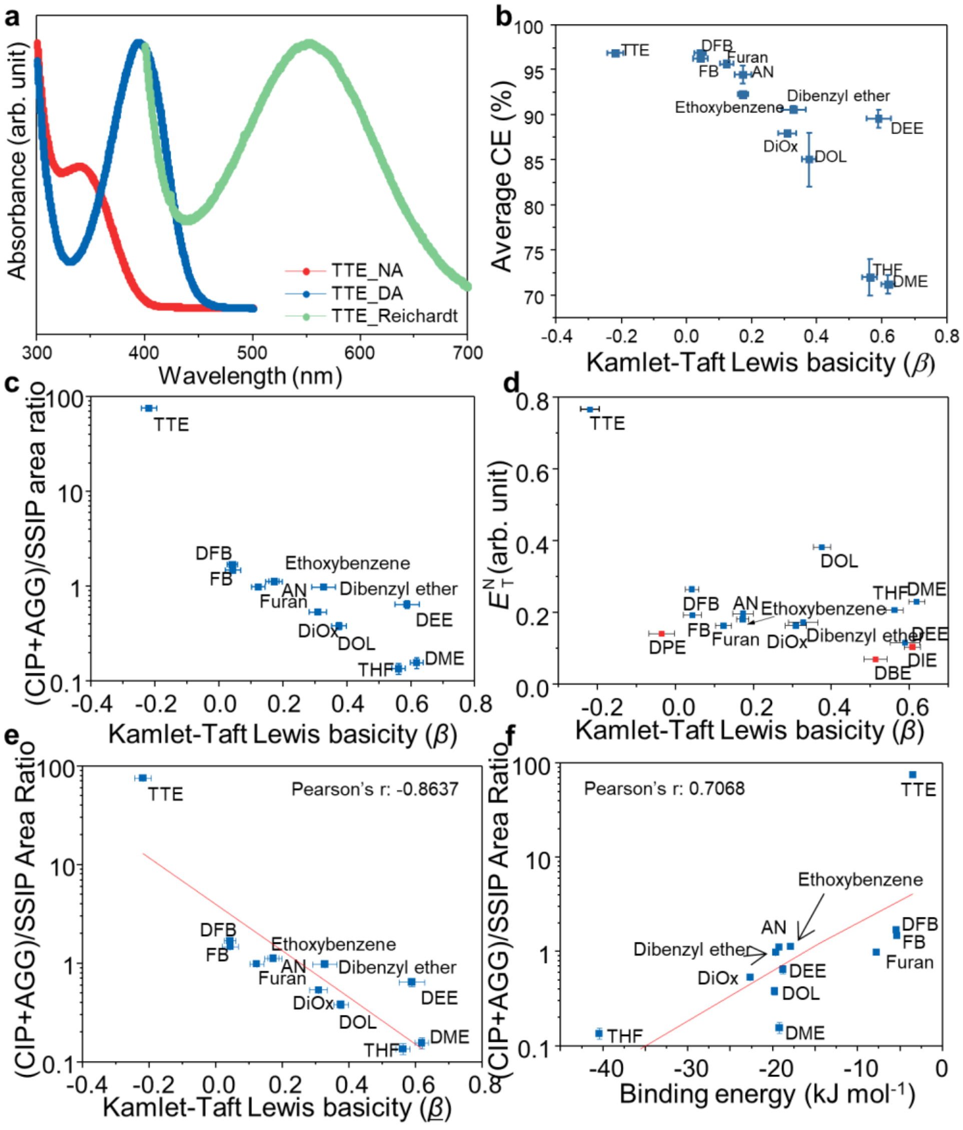

Supplementary Figure 9. **Applying the performance of conventional FNSC (TTE) to solvatochromic parameters. a** Spectra of dyes (NA, DA, Reichardt's dye) in TTE solvent. **b** Correlation of Kamlet-Taft Lewis basicity (β) with average CE. **c** Correlation of β with (CIP+AGG)/SSIP area ratio and **d** final two-axis plot (β vs E_T^N). Correlation plot of (CIP+AGG)/SSIP area ratio with **e** Kamlet-Taft Lewis basicity (β) and **f** binding energy (solvent model). Error bars shown here are 95% CI.

Note: Because of large deviation of (CIP+AGG)/SSIP area ratio of TTE with other organic molecules, standard scale like Fig. 1c would not offer a fair comparison. To compare the relative position of TTE, we chose log scale for (CIP+AGG)/SSIP area ratio. Binding energy vs (CIP+AGG)/SSIP plot was also modified to log scale. Two figures (Supplementary Figure 9e-f**)** with log scale confirm that our parameter (β) shows higher correlation than calculated binding energy under solvent model (EC:DEC=1:1 (v/v)).

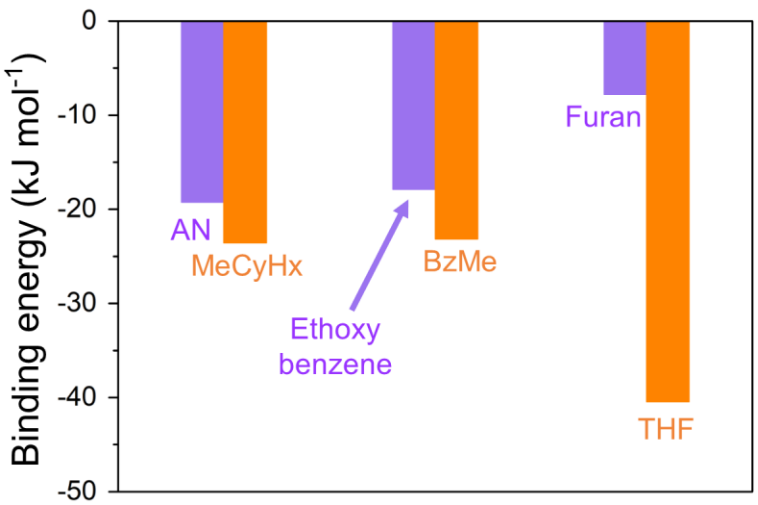

Supplementary Figure 10. The calculated binding energy of lithium ion under solvent model. We used EC:DEC=1:1 v/v as the model.

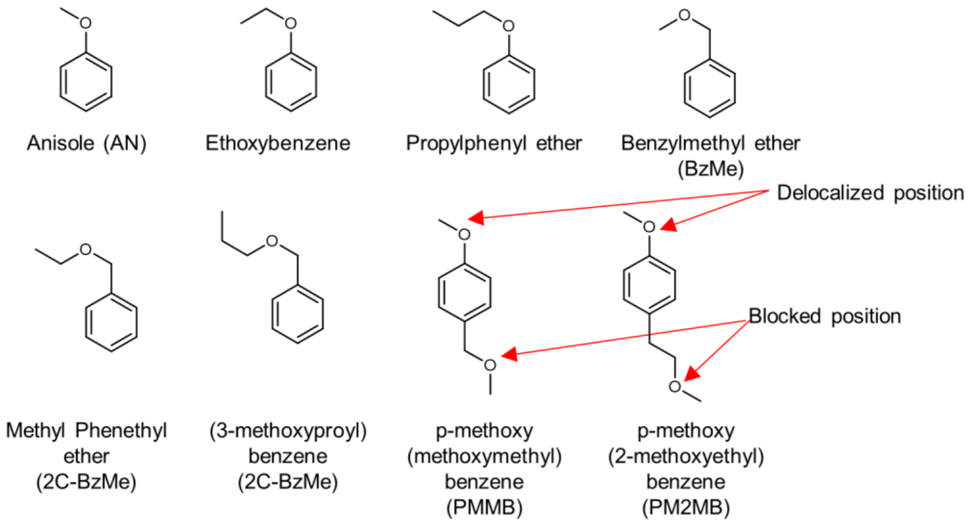

Supplementary Figure 11. **The names and the abbreviations of molecules studied in Supplementary Note 5**. Especially, the two oxygen atoms of PMMB and PM2MB are distinguished as delocalised and blocked.

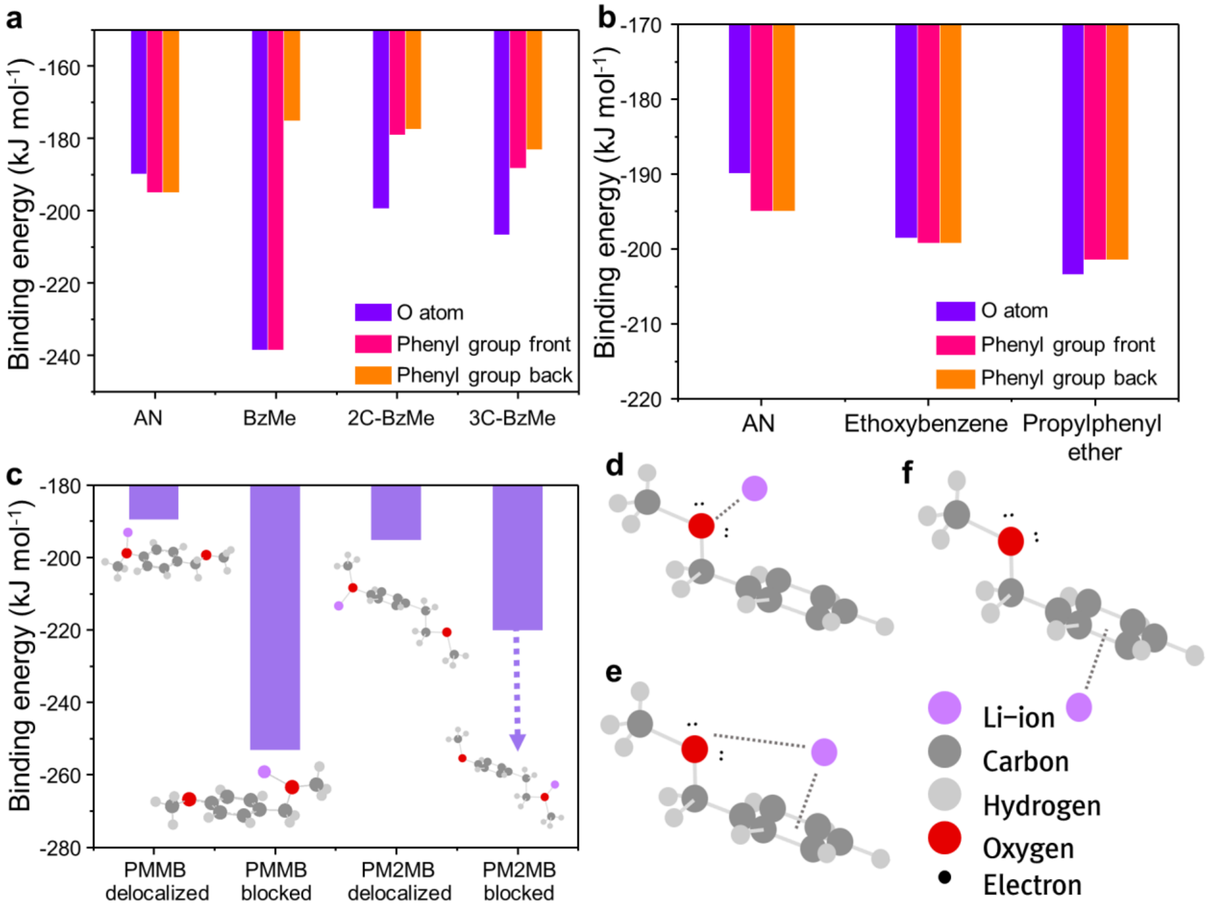

Supplementary Figure 12. The binding energies of lithium ion with molecules in Supplementary Figure 11. a AN, BzMe, (2C-BzMe) and 3C-BzMe **b** AN, ethoxybenzene and propylphenyl ether **c** different oxygen atoms in PMMB and PM2MB. Binding configuration of lithium ion with representative BzMe to **d** O atom **e** phenyl group front and **f** phenyl group back. All calculations were performed under vacuum model.

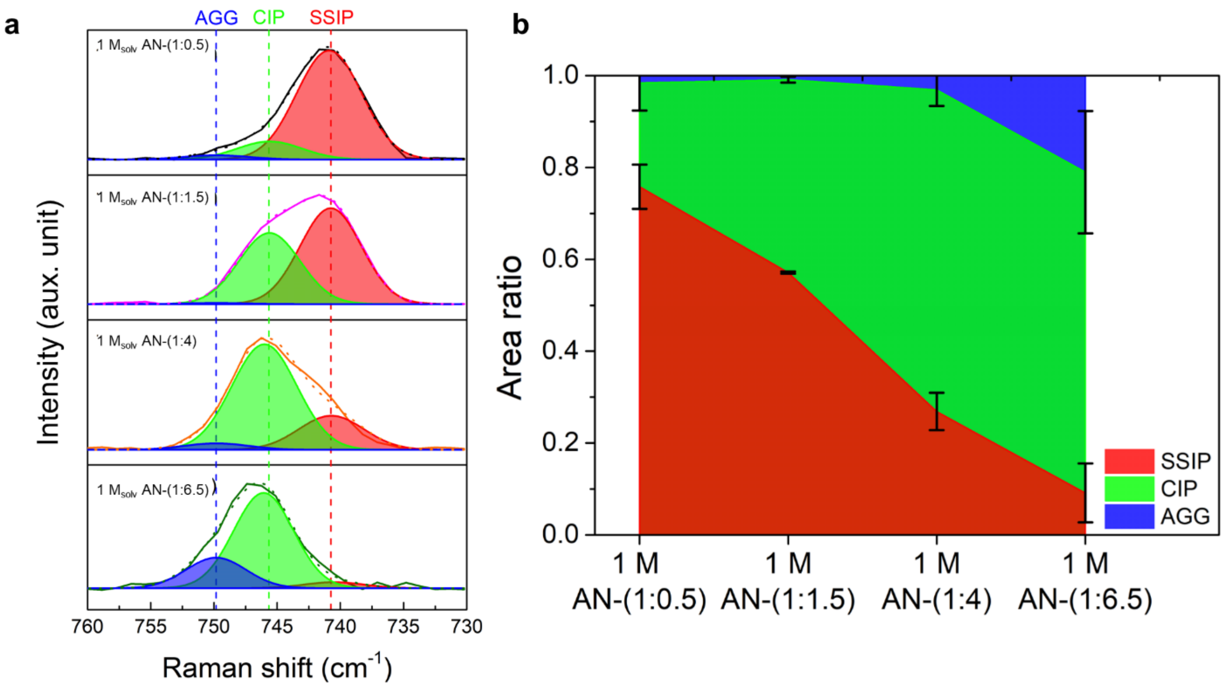

Supplementary Figure 13. **Changing solvation structure of electrolytes varying the volume ratio of AN. a** Raman peak deconvolution for TFSI$^-$ shift with increasing amount of AN and **b** and the ratios of SSIP, CIP and AGG for each electrolyte. Error bars shown here are 95% CI.

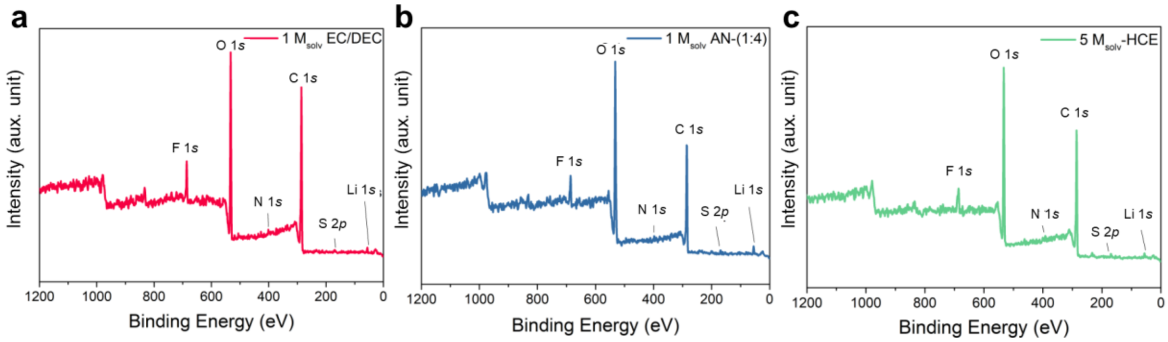

Supplementary Figure 14. XPS survey spectra of SEI layer on Cu electrode after 10 cycles 0.5 mAh cm^{-2} at 0.5 mA cm^{-2}. a 1 M_{solv} EC/DEC **b** 1 M_{solv} AN-(1:4) and **c** 5 M_{solv}-HCE

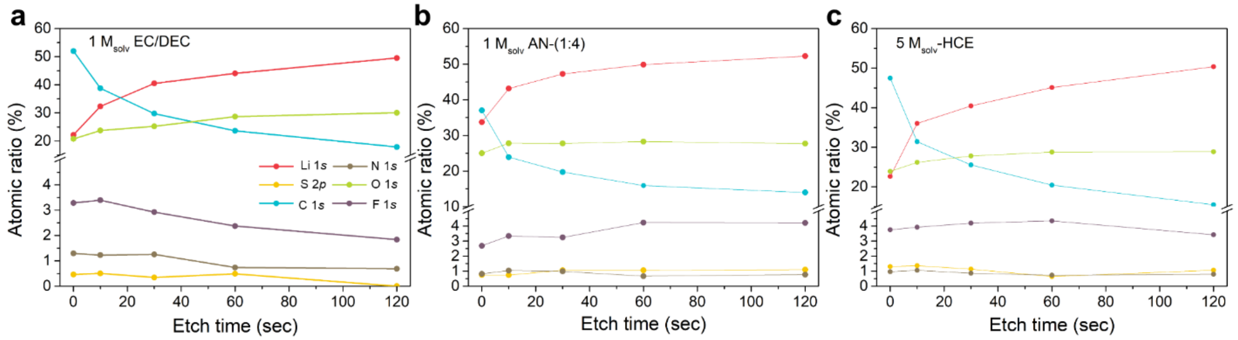

Supplementary Figure 15. XPS atomic ratio of SEI layer on Cu electrode after 10 cycles for 0.5 mAh cm^{-2} at 0.5 mA cm^{-2}. a 1 M_{solv} EC/DEC electrolyte (**b**) 1 M_{solv} AN-(1:4) and **c** 5 M_{solv}-HCE.

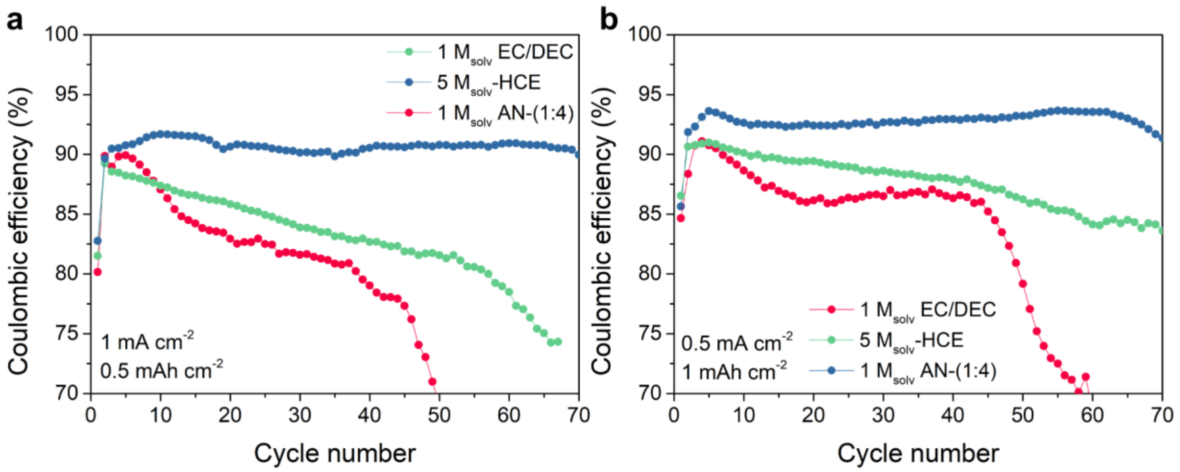

Supplementary Figure 16. **Cycling Performance of Li|Cu cells using different electrolytes at various current and capacity density. a** 1 mA cm^{-2} to 0.5 mAh cm^{-2} and **b** 0.5 mA cm^{-2} to 1 mAh cm^{-2}

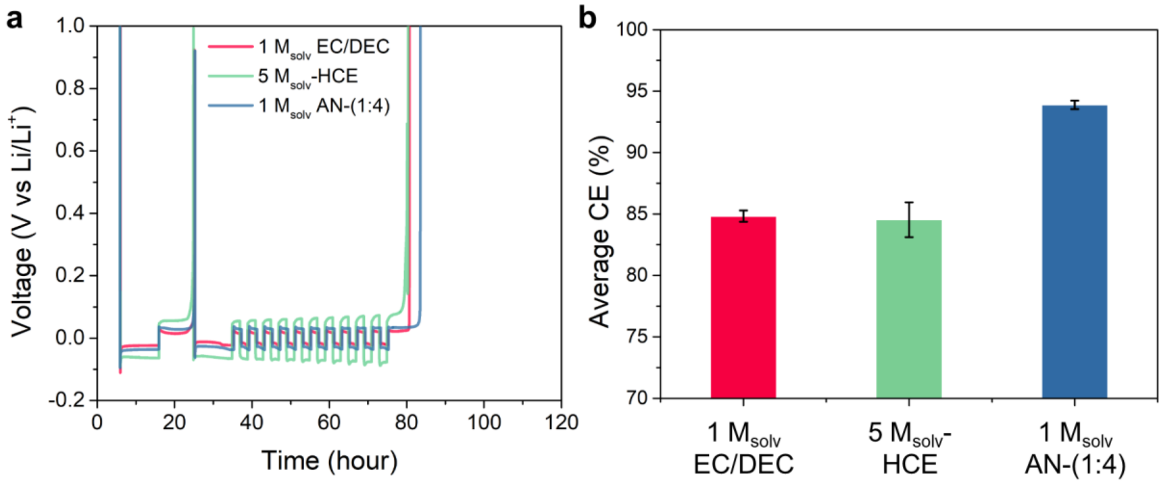

Supplementary Figure 17. **Average CE of 1 M$_{solv}$ EC/DEC, 5 M$_{solv}$-HCE, and 1 M$_{solv}$ AN-(1:4). a** Time vs voltage graph of the experiment measuring average CE and **b** average CE value obtained from the modified Aurbach method. Throughout the process, the current density was fixed at 0.5 mA cm^{-2}. The initial precycle (deposition and stripping) was performed to 5 mAh cm^{-2} then followed by deposition of 5 mAh cm^{-2} lithium (Q_R). After 10 deposition and stripping cycles to 1 mAh cm^{-2}, final stripping (1 V cutoff) was done (Q_S). Error bars shown here are 95% CI.

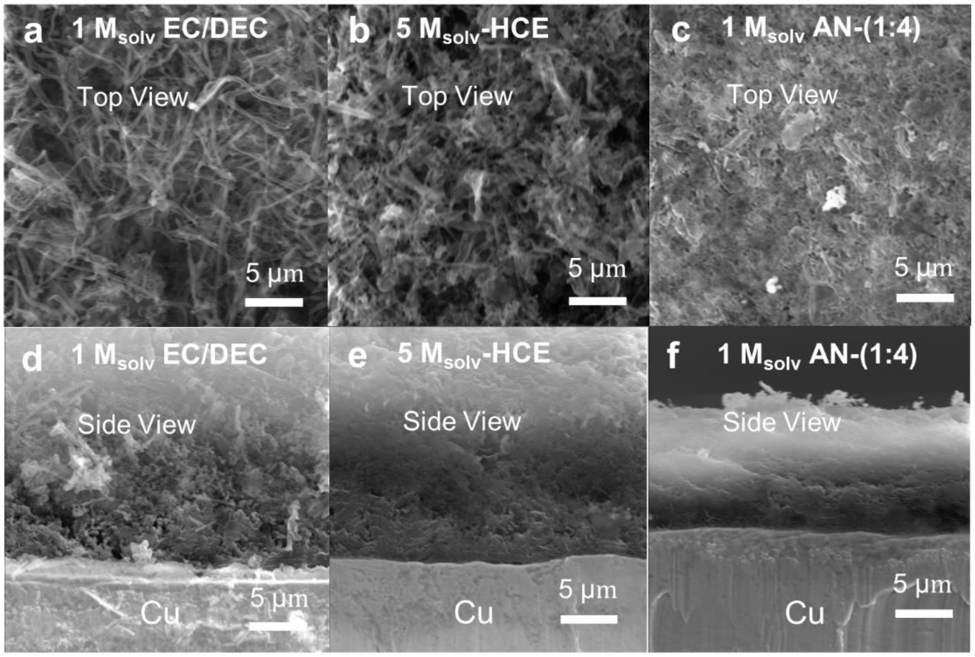

Supplementary Figure 18. **Magnified SEM images of deposited lithium metal. a-c** Top-down SEM images of lithium deposition on Cu foil after 10th depostion cycle at 0.5 mA cm^{-2} to 0.5 mAh cm^{-2} using different electrolytes and **d-f** SEM images of the cross section of the same samples, respectively.

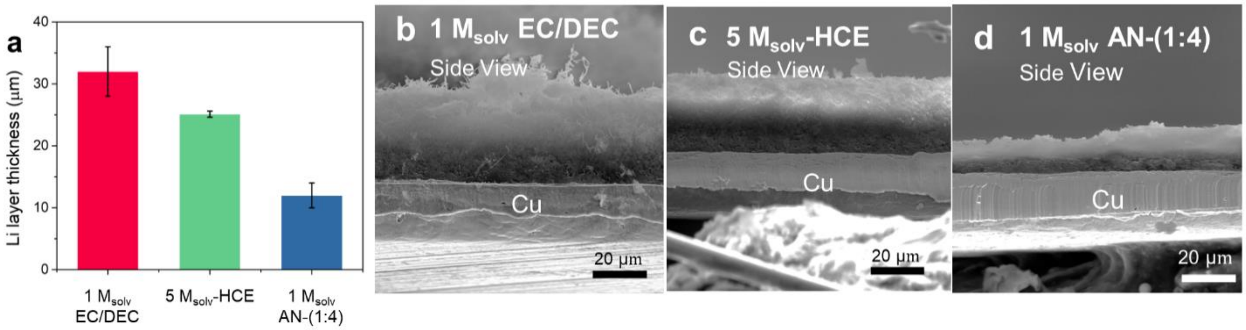

Supplementary Figure 19. **Thickness of deposited lithium metal. a** The thickness of lithium metal deposited on the Cu after the 10th deposition cycle at 0.5 mA cm^{-2} to 0.5 mAh cm^{-2}. SEM images of the cross section of the **b** 1 M_{solv} EC/DEC, **c** 5 M_{solv}-HCE, and **d** 1 M_{solv} AN-(1:4) used for calculating lithium layer thickness in Fig. S19a. Error bars shown here are 95% CI.

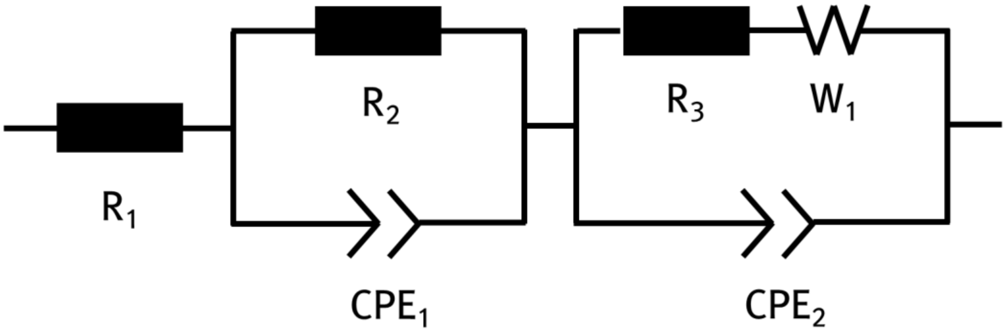

Supplementary Figure 20 The equivalent circuit used for the EIS Nyquist plot fitting.

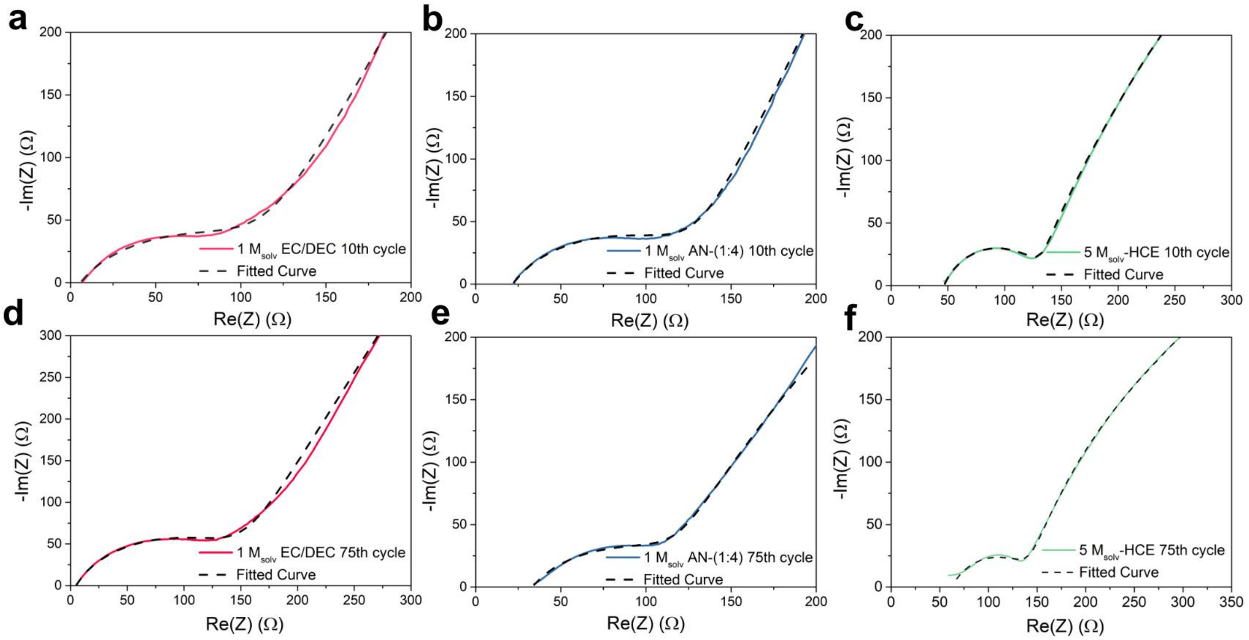

Supplementary Figure 21. Nyquist plot of EIS data and its fitting curve obtained for each electrolyte.

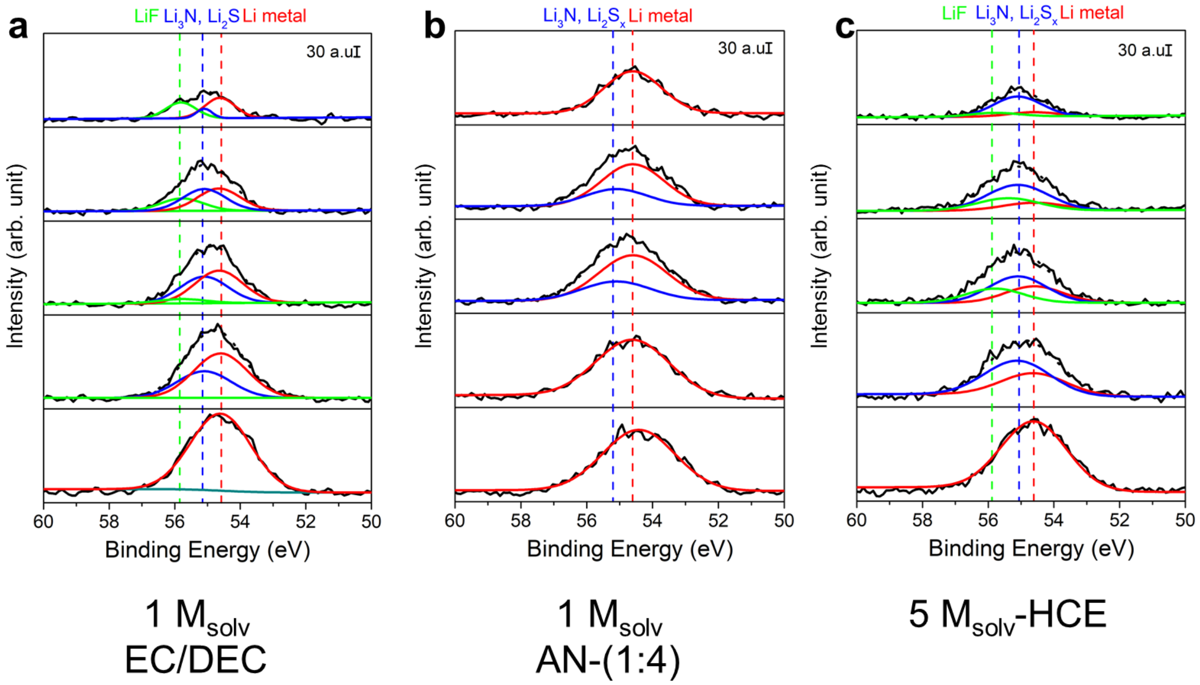

Supplementary Figure 22. Li 1*s* spectra of Cu electrode at different etching times after 10 cycles (0.5 mA cm^{-2}, 0.5 mAh cm^{-2}, charged sample). **a** 1 M_{solv} EC/DEC, **b** 1 M_{solv} AN-(1:4), and **c** 5 M_{solv}-HCE

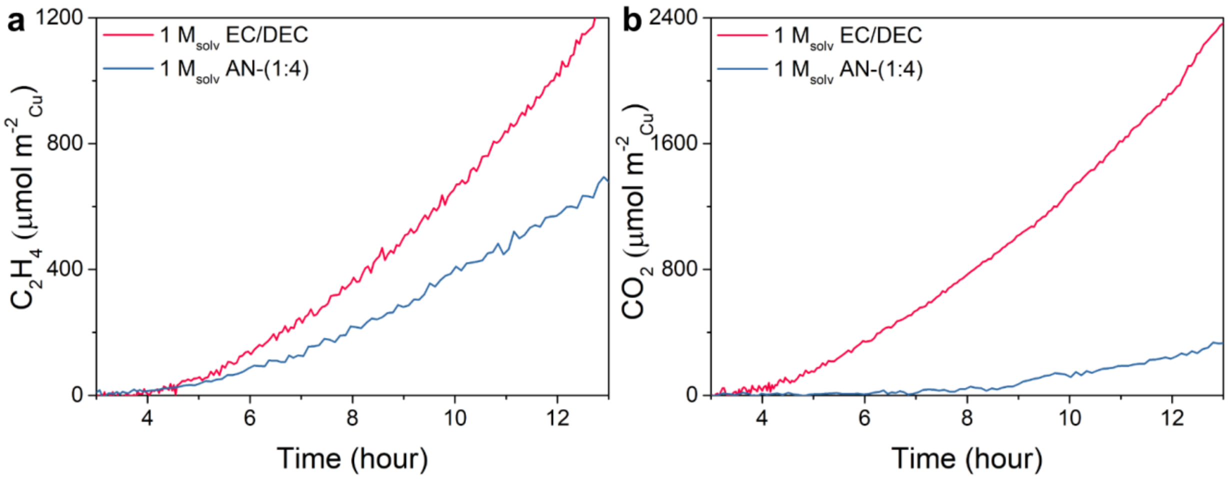

Supplementary Figure 23. **Gas evolution profile obtained from DEMS. a** C_2H_4 and **b** CO_2

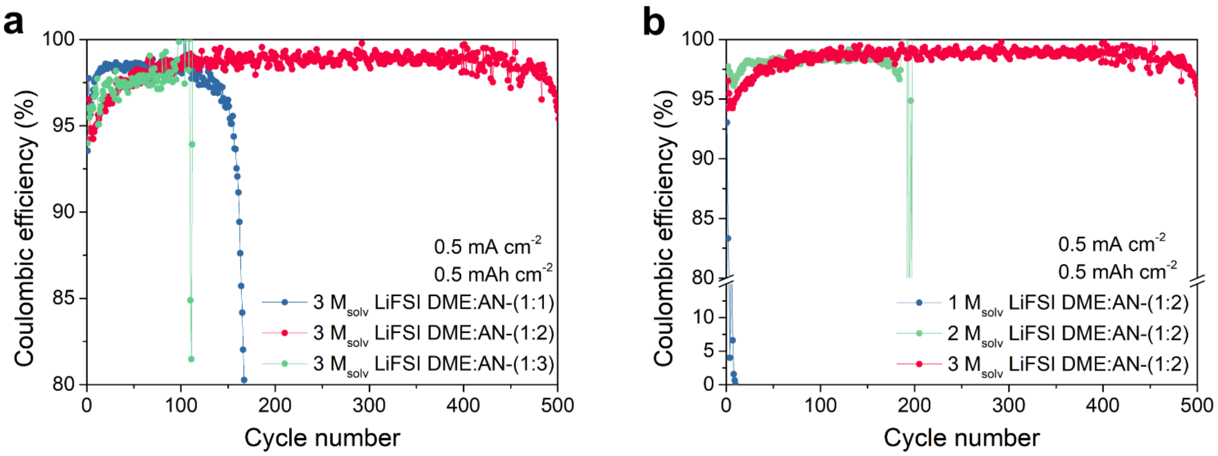

Supplementary Figure 24. **Cycling performance of Li|Cu cells cycled at 0.5 mA cm^{-2} to 0.5 mAh cm^{-2} under different AN-containing electrolytes. a** Containing different volume fraction of AN in 3 M_{solv} LiFSI DME system and **b** different salt concentrations in DME:AN-(1:2 v/v) system.

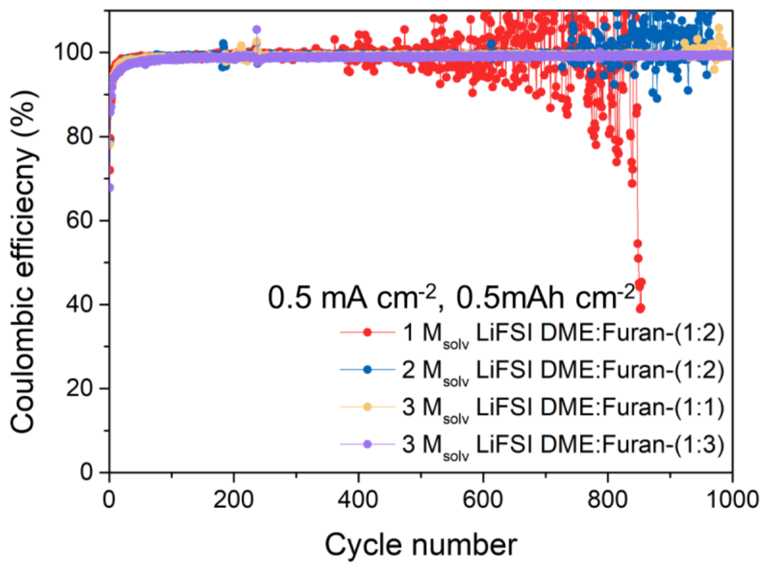

Supplementary Figure 25. Cycling performance of Li|Cu cells cycled at 0.5 mA cm^{-2} to 0.5 mAh cm^{-2} with various concentration of LiFSI and DME to furan volumetric ratio.

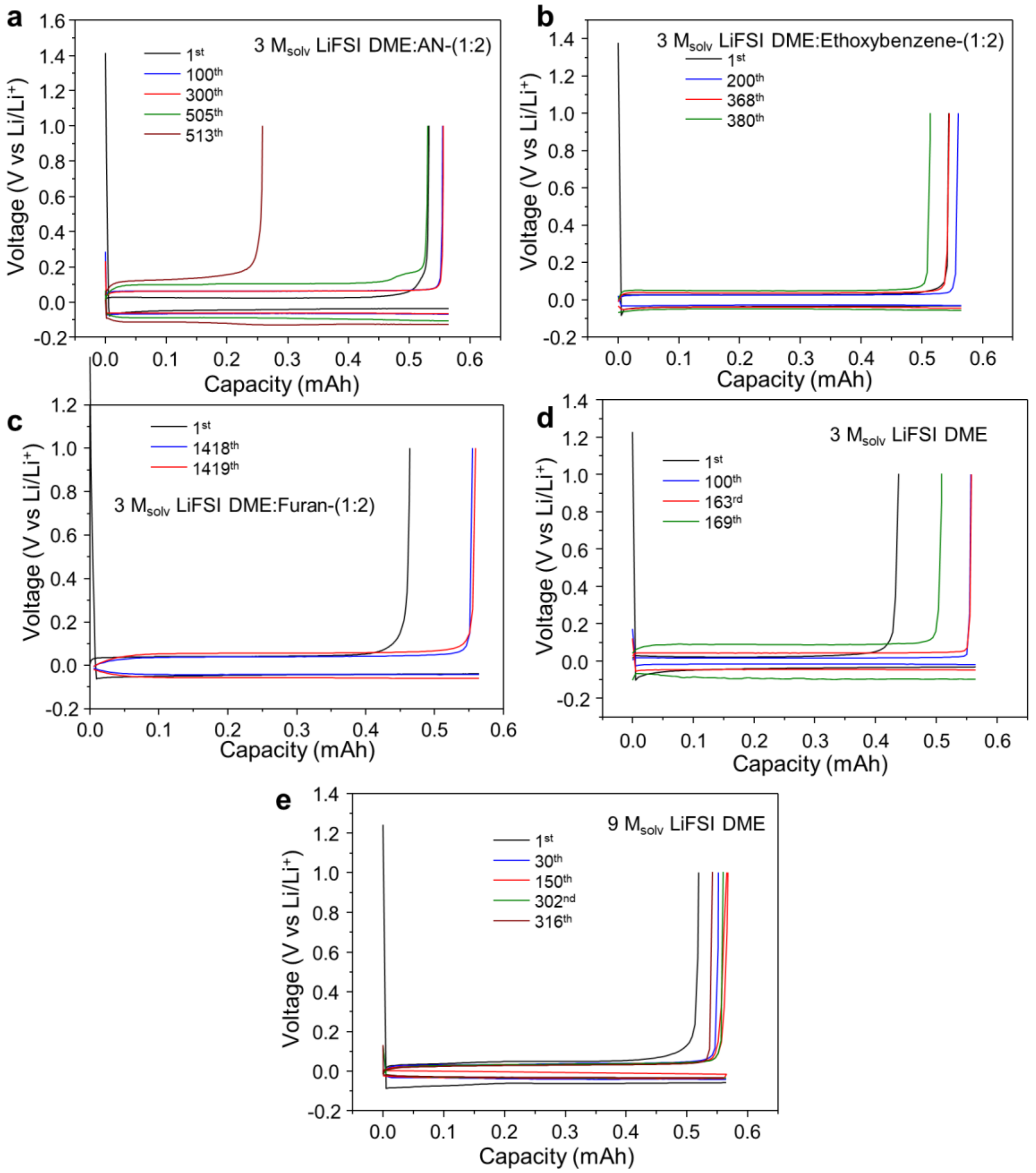

Supplementary Figure 26. Capacity-voltage profiles of the Li|Cu cells cycled at 0.5 mA cm^{-2} to 0.5 mAh cm^{-2}. a 3 M_{solv} LiFSI DME:AN-(1:2) **b** 3 M_{solv} LiFSI DME:Ethoxybenzene-(1:2) **c** 3 M_{solv} LiFSI DME:Furan-(1:2) **d** 3 M_{solv} LiFSI DME and **e** 9 M_{solv} LiFSI DME. Refer to Fig. 4a for the corresponding CE graphs for each electrolyte.

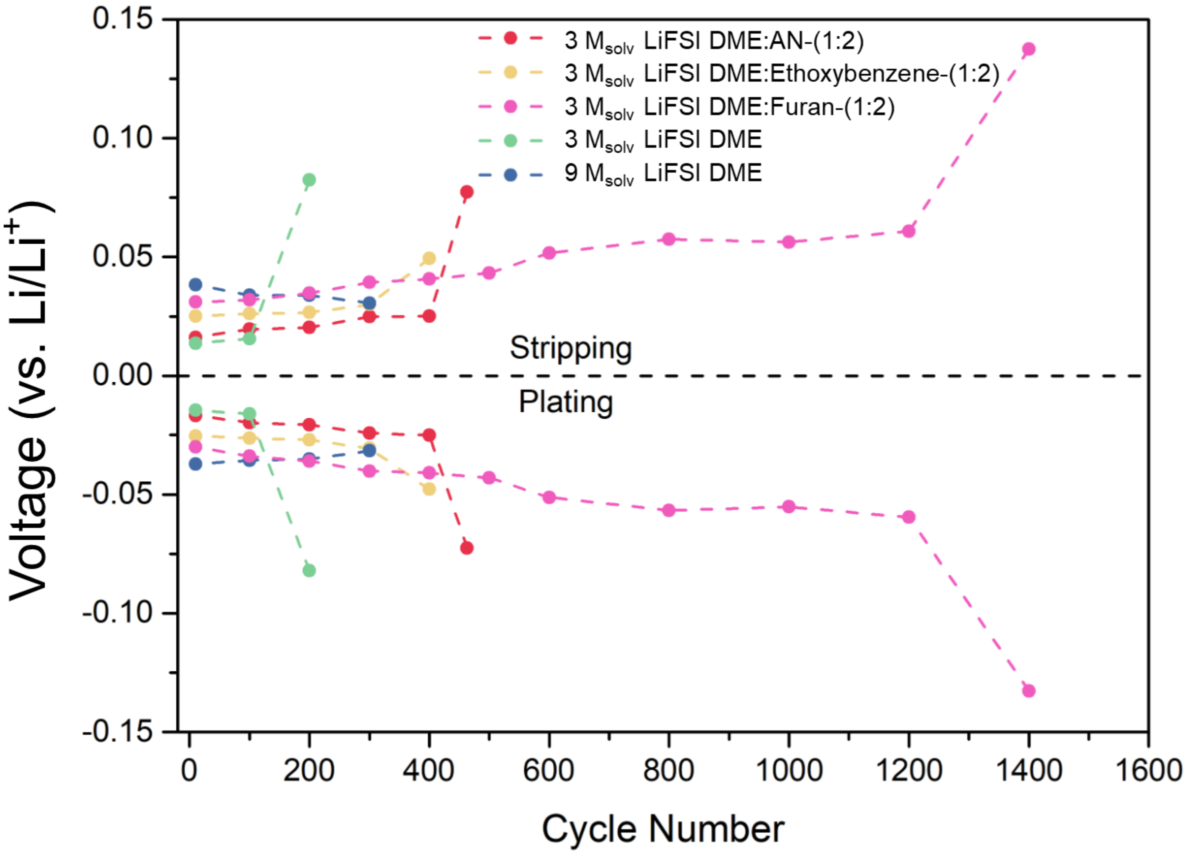

Supplementary Figure 27. Overpotential profile of various electrolytes under cycling at 0.5 mA cm^{-2} to 0.5 mAh cm^{-2}.

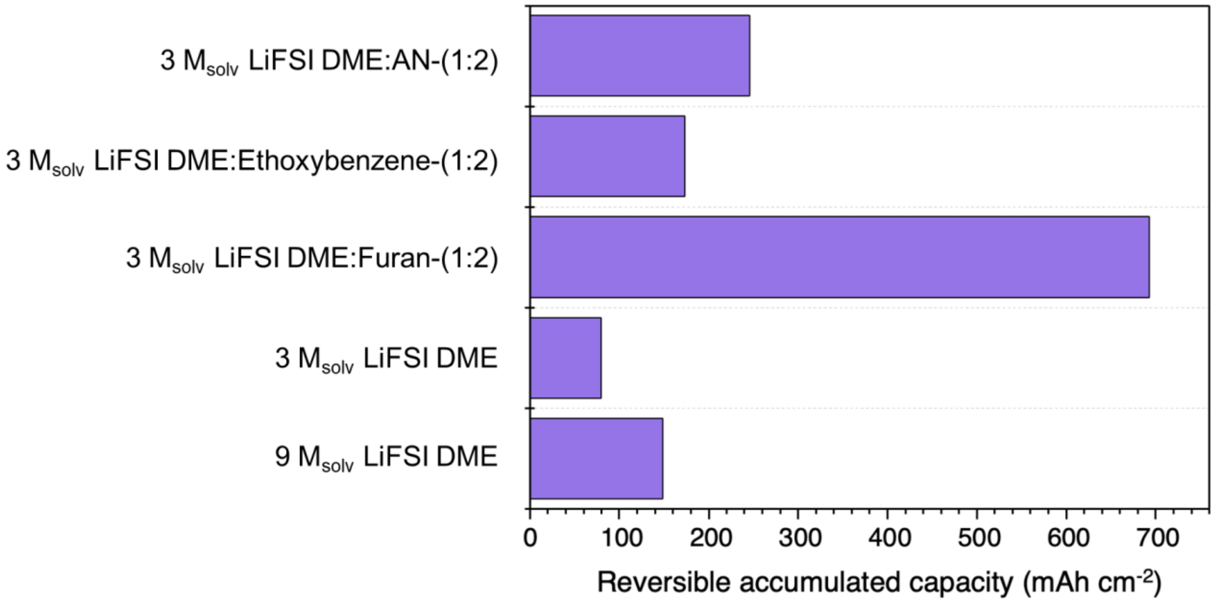

Supplementary Figure 28. Reversible accumulated capacity of Li|Cu cell calculated from Fig. 4a.

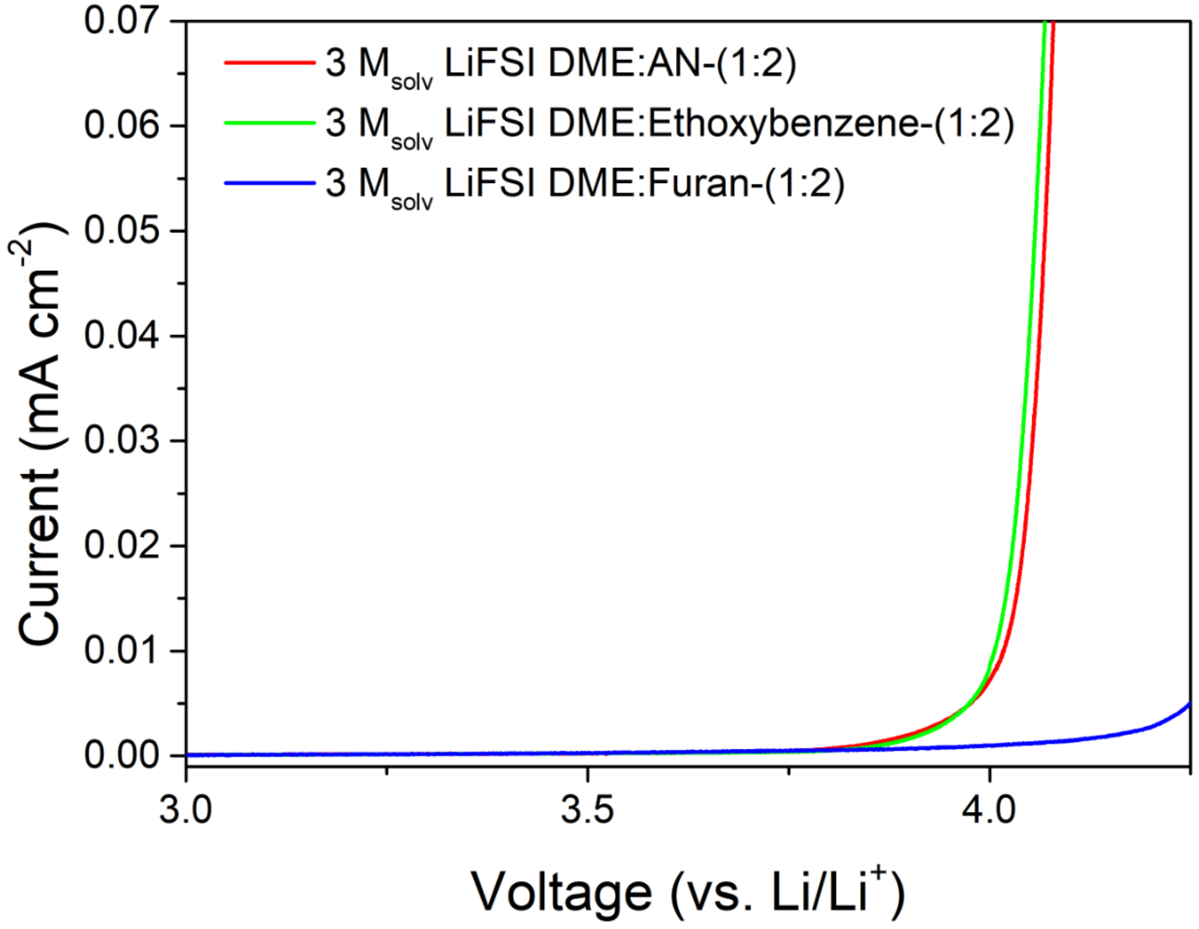

Supplementary Figure 29. Oxidative stability of NFNSC-containing electrolytes

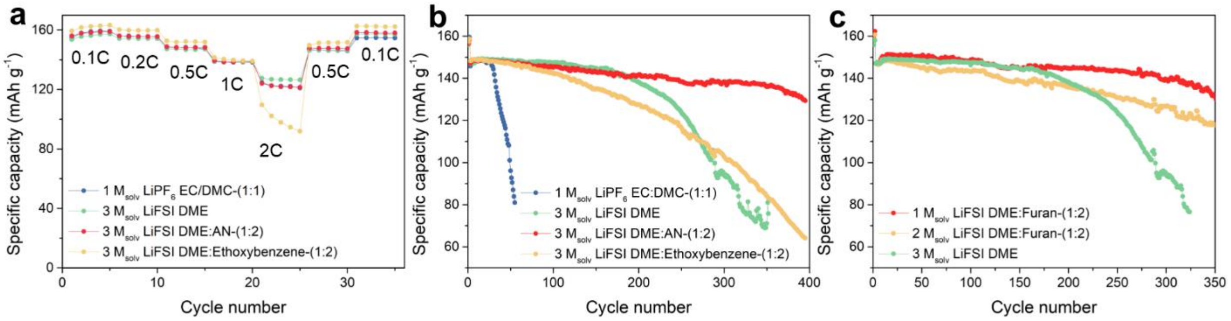

Supplementary Figure 30. **LFP full cell experiments with mass loading of ~ 11 mg cm^{-2}. a** Rate test of thick Li (300 μm)|LFP full cell. **b** Cycling test of electrochemically deposited thin-foil Li|LFP full cell. **c** Cycling test of furan-containing electrolyte with decreased salt concentration. The experiments were performed under the same configuration as in Supplementary Figure 30b. Experimental condition for rate test is denoted in Supplementary Figure 30a and the cycling experiments were done under 2 cycles of 0.1 C formation cycle and cycling with 0.5 C.

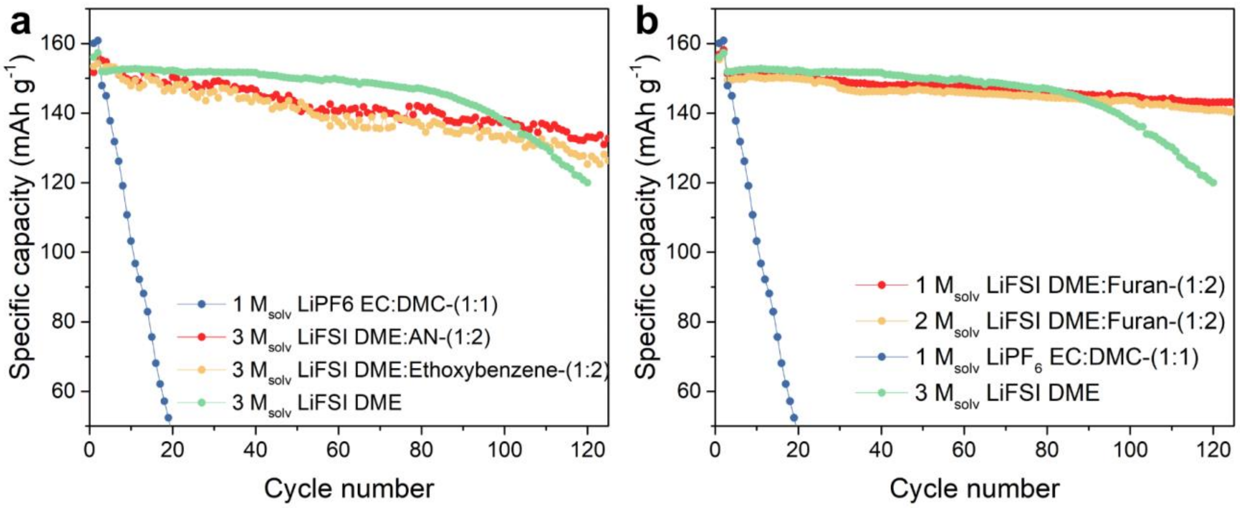

Supplementary Figure 31. LFP full cell experiments with mass loading of ~ 21 mg cm^{-2} (N/P ratio ~1.6). a Cycling test of electrochemically deposited thin-foil Li|LFP full cell. **b** Cycling test of furan-containing electrolyte with decreased salt concentration. The experiments were performed under the same configuration as in Supplementary Figure 31a**.** Cycling experiments were done under 2 cycles of 0.05 C formation cycle and cycling with 0.2 C.

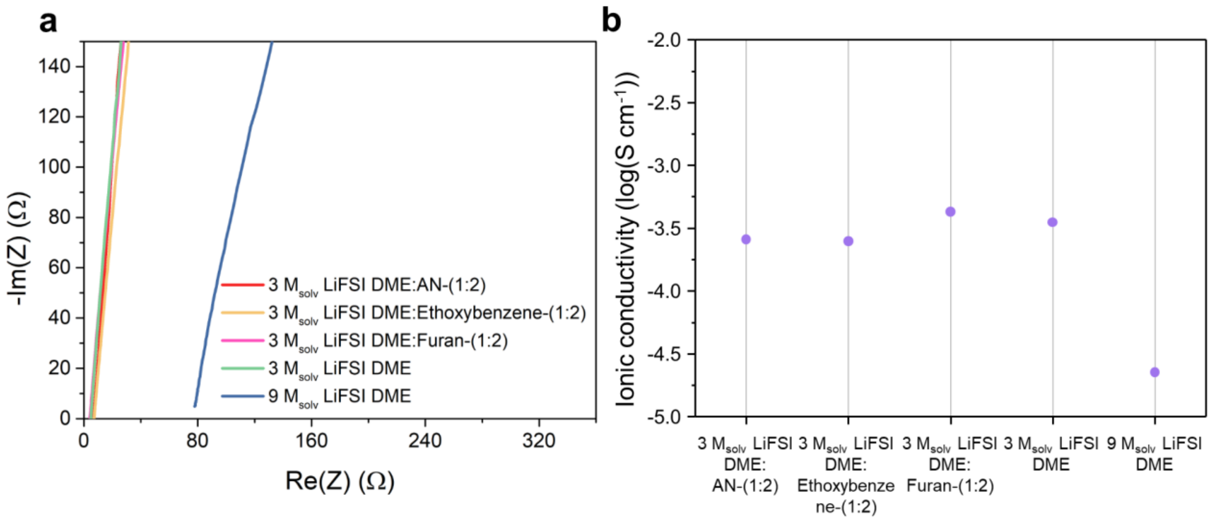

Supplementary Figure 32. **Ionic conductivity of various electrolytes studied in Fig. 4. a** Electrochemical impedance spectra of SS|SS cells (SS for stainless steel) with different electrolytes at room temperature and **b** log of ionic conductivity obtained using the resistance in Supplementary Figure 32a.

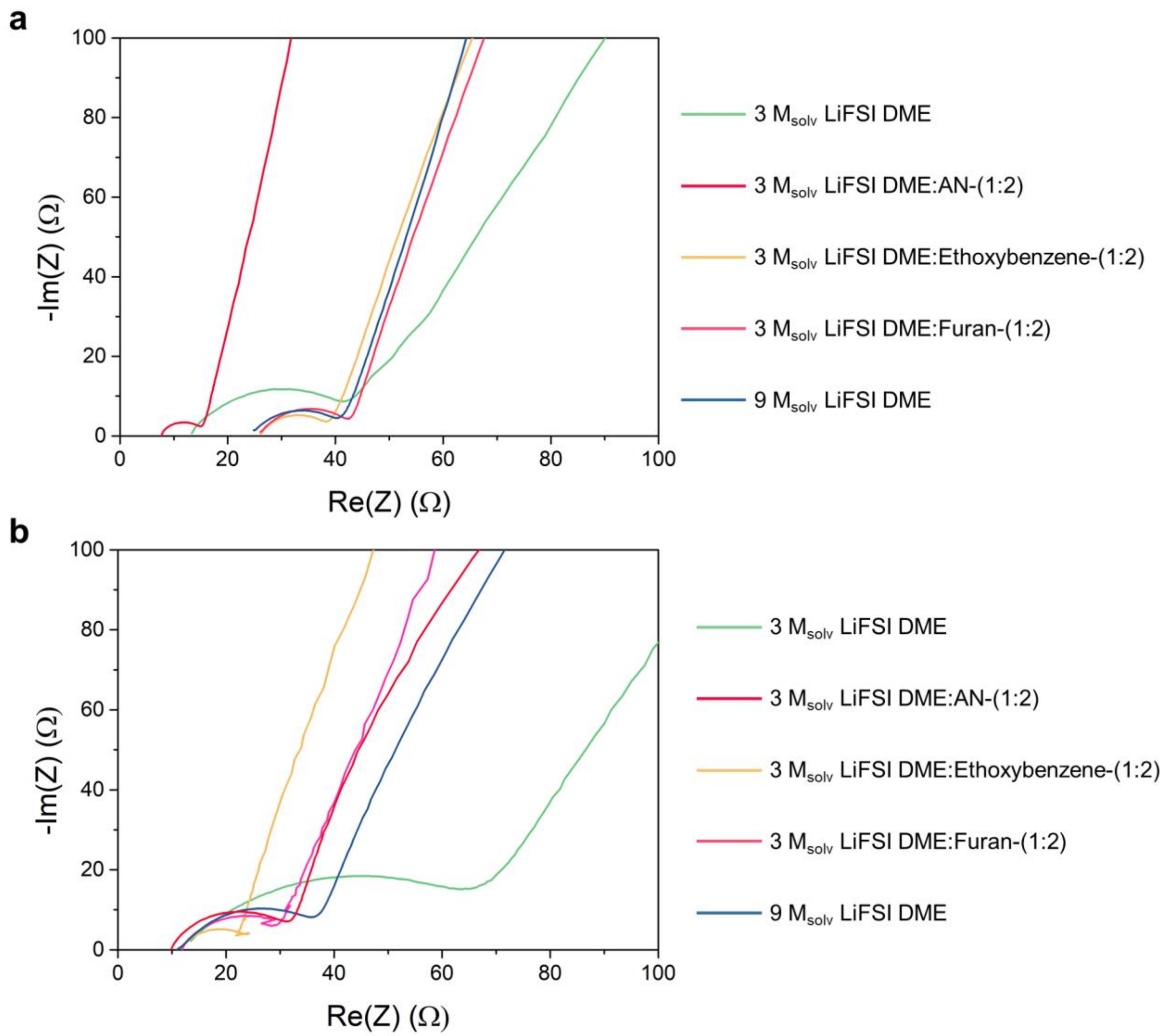

Supplementary Figure 33. EIS Nyquist plots of Li|Cu cells after cycling. a 1st cycle and **b** 55th cycle at 0.5 mA cm^{-2} to 0.5 mAh cm^{-2}.

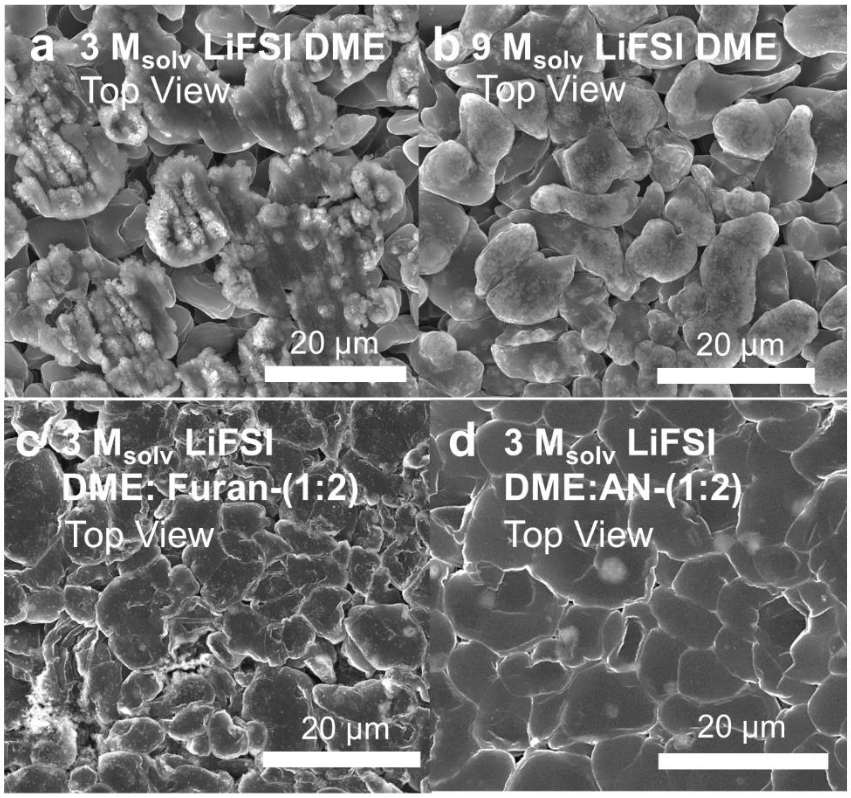

Supplementary Figure 34. Top-down SEM images of deposited lithium on Cu foil (0.5 mA cm^{-2}, 0.5 mAh cm^{-2}). **a** 3 M_{solv} LiFSI DME, **b** 9 M_{solv} LiFSI DME, **c** 3 M_{solv} LiFSI DME:Furan-(1:2), and **d** 3 M_{solv} LiFSI DME:AN-(1:2).

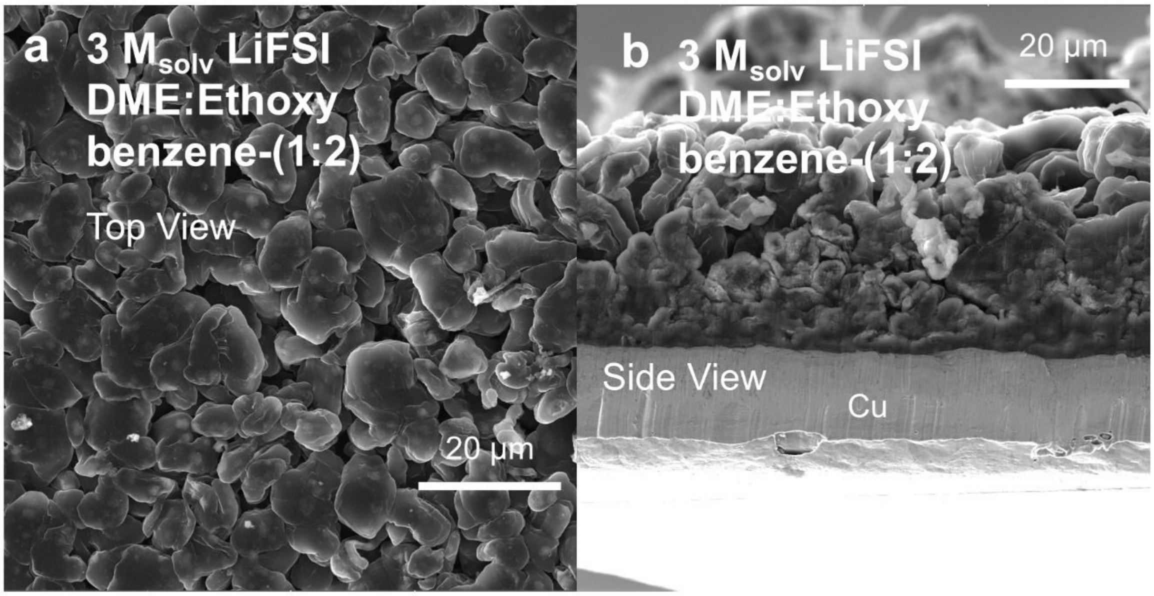

Supplementary Figure 35. SEM images of deposited lithium on Cu foil in 3 M_{solv} LiFSI DME:Ethoxybenzene-(1:2), (0.5 mA cm^{-2}, 0.5 mAh cm^{-2}). **a** Top-down and **b** cross-section SEM images of deposited lithium on Cu foil in 3 M_{solv} LiFSI DME:Ethoxybenzene-(1:2).

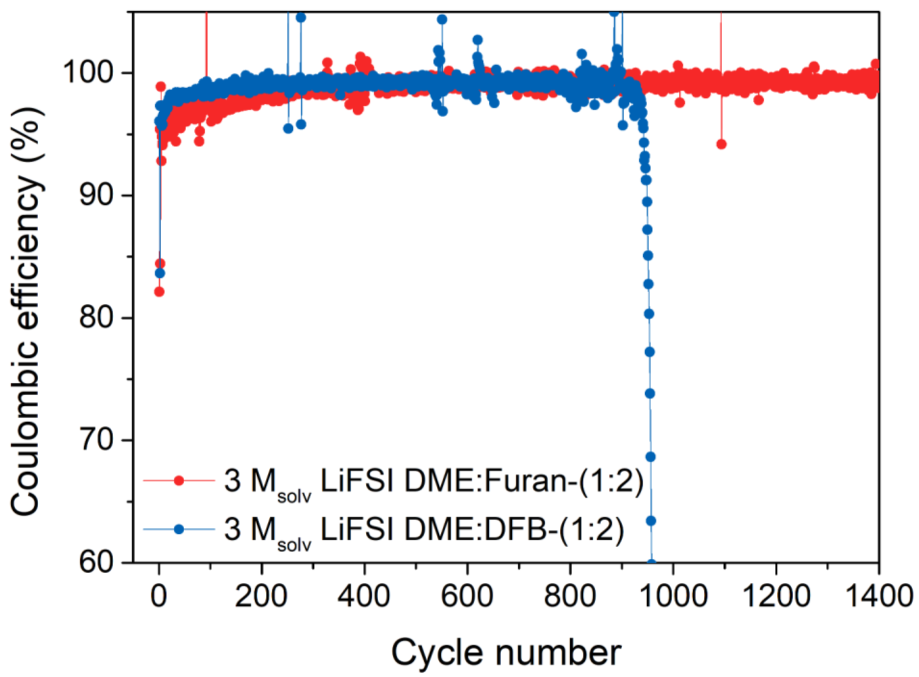

Supplementary Figure 36. Cycling performance of Li|Cu cell with different electrolytes at 0.5 mA cm^{-2} to 0.5 mAh cm^{-2}.

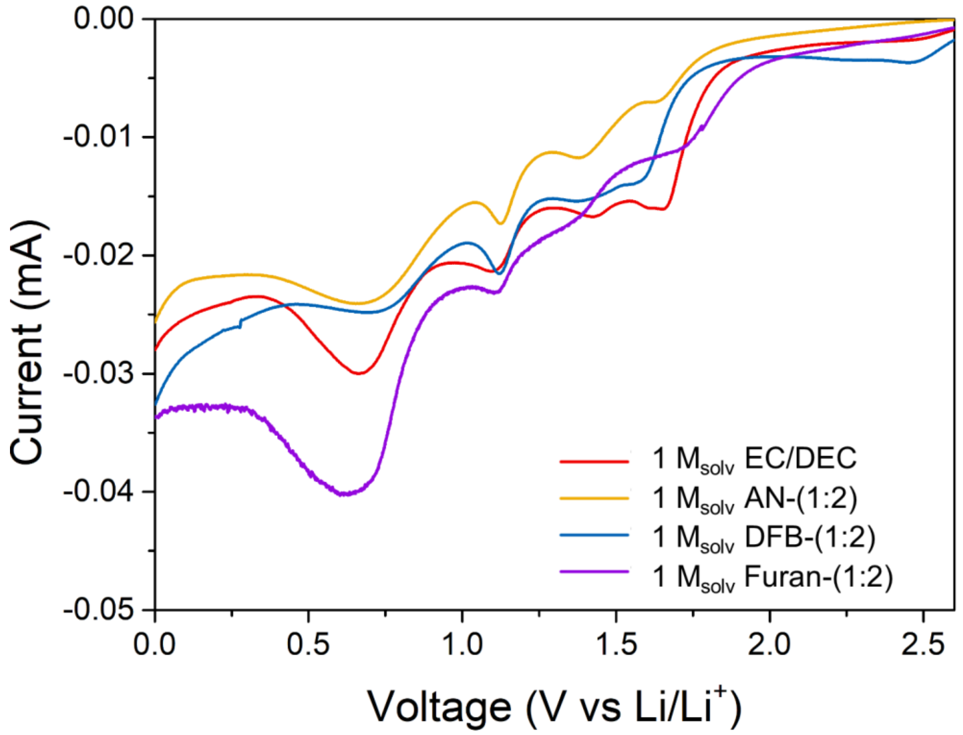

Supplementary Figure 37. LSV profiles of Li|Cu cells consisting different electrolytes at a scan rate of 1 mV s^{-1}.

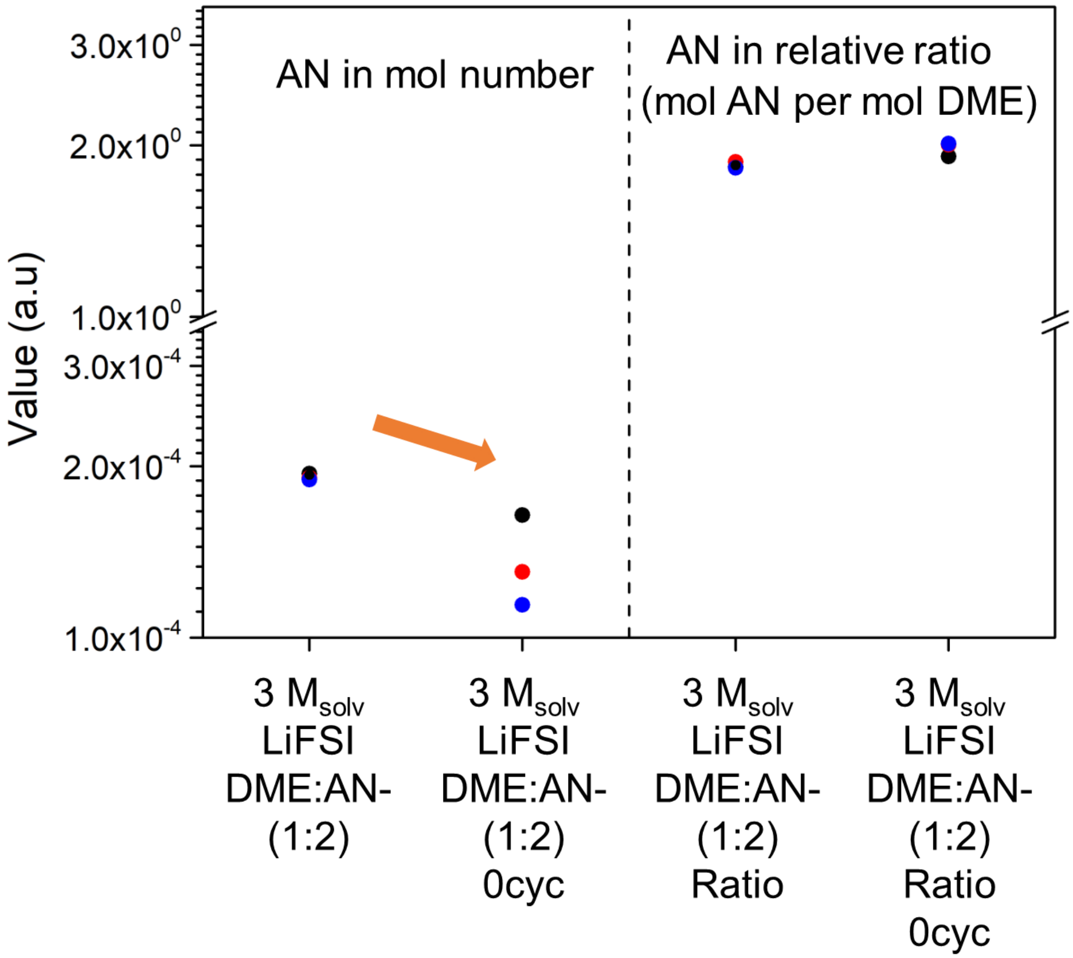

Supplementary Figure 38. Remaining quantity of AN in mol number and in relative ratio compared to DME. We quantified the AN from pristine 40 μL electrolytes and from disassembled coin cell (denoted as 0 cyc).

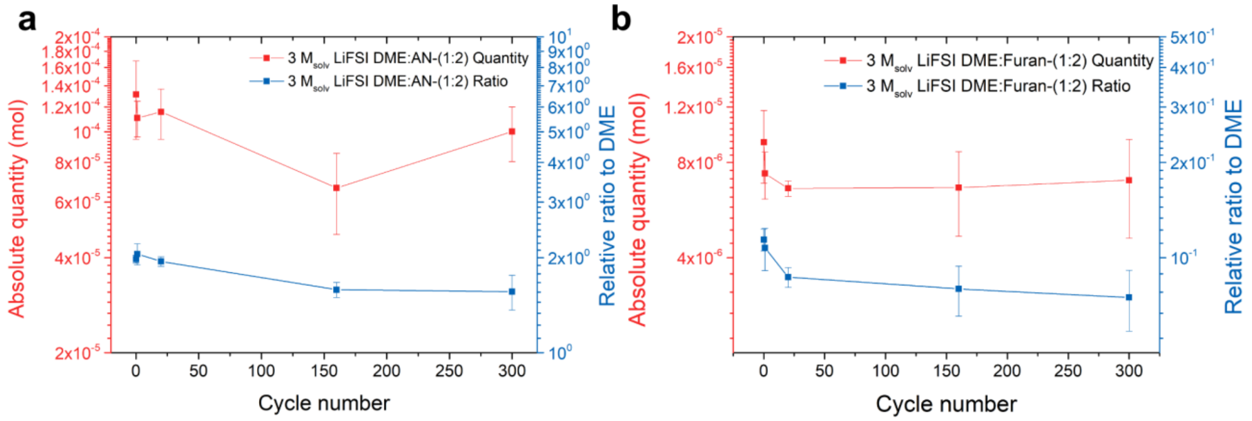

Supplementary Figure 39. Change in remaining quantity of NFNSCs after different number of cycles. a AN and **b** furan. Larger error bars are observed for values expressed in the absolute quantity. The seemingly larger deviation of furan might be attributed to its low boiling point (31.3 ℃) compared to AN (154 ℃). Error bars shown here are 95% CI.

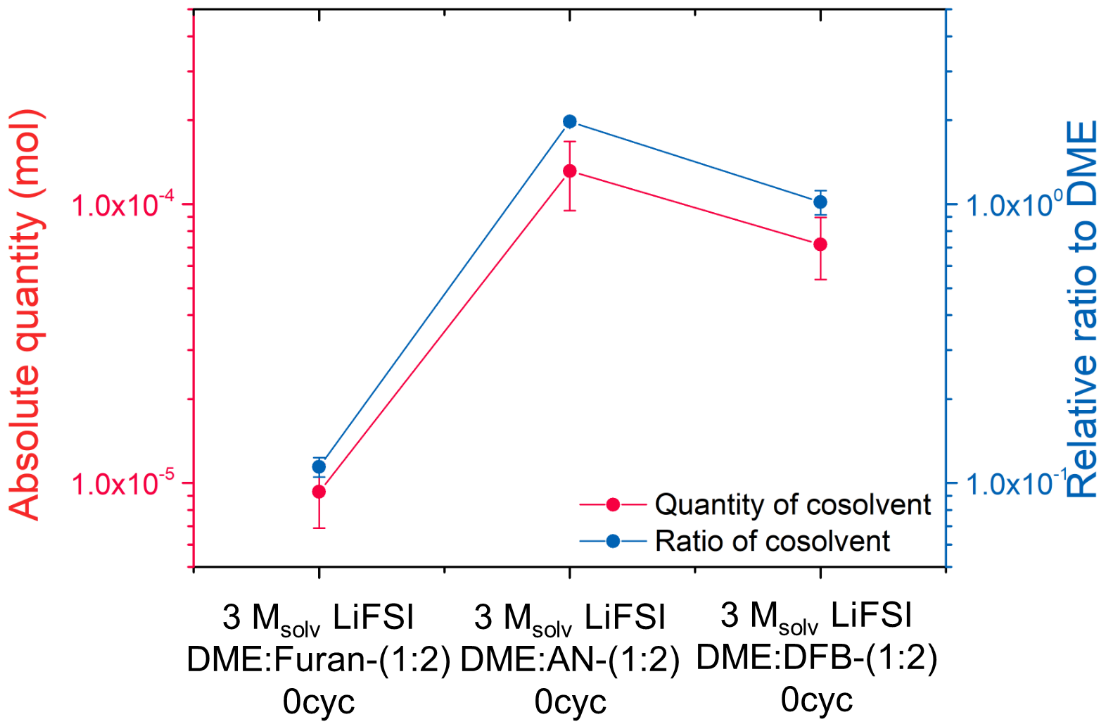

Supplementary Figure 40. Absolute quantity and relative ratio of furan, AN, and DFB in electrolytes extracted from disassembled coin cells before the cycling test. Larger error bars are observed for values expressed in the absolute quantity. Error bars shown here are 95% CI.

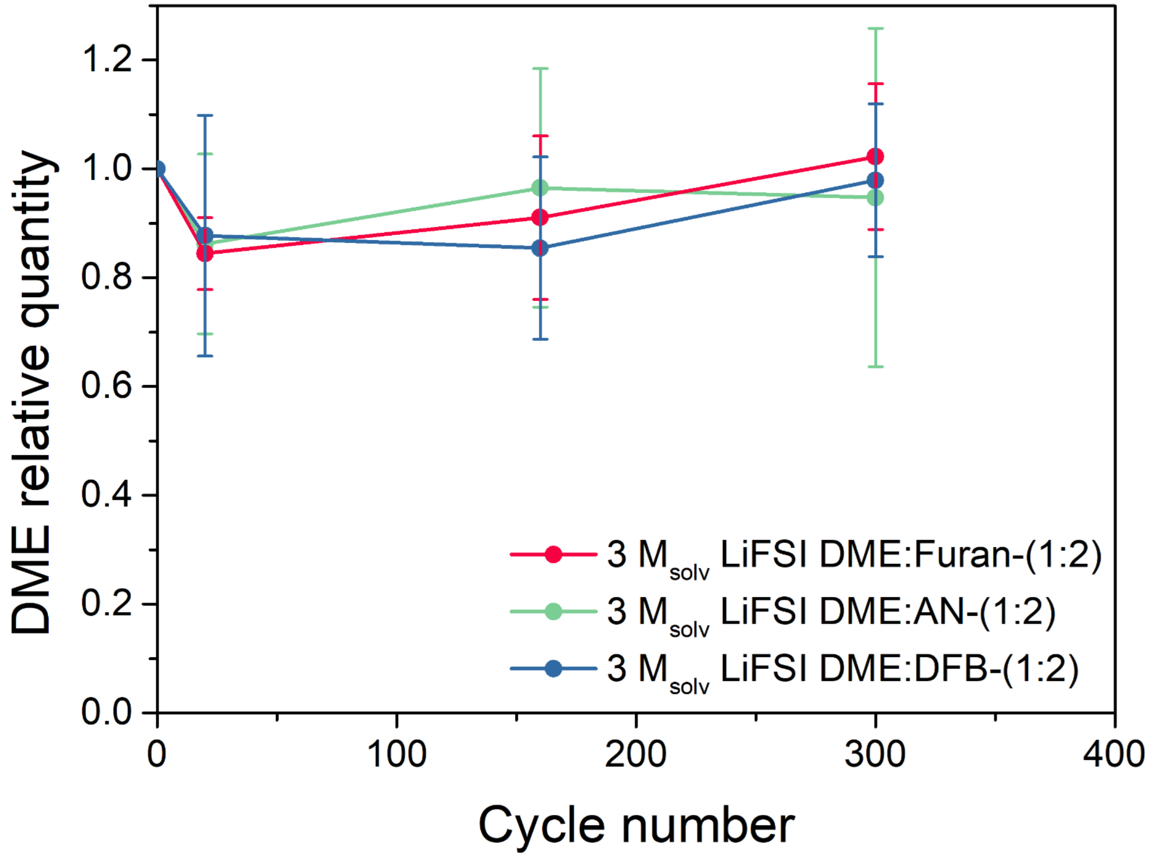

Supplementary Figure 41. Remaining quantity of DME relative to that of 0 cycle in Li|Cu coin cells disassembled after the different number of cycles. The cells were cycled at 0.5 mA cm^{-2} to 0.5 mAh cm^{-2}. Error bars shown here are 95% CI.

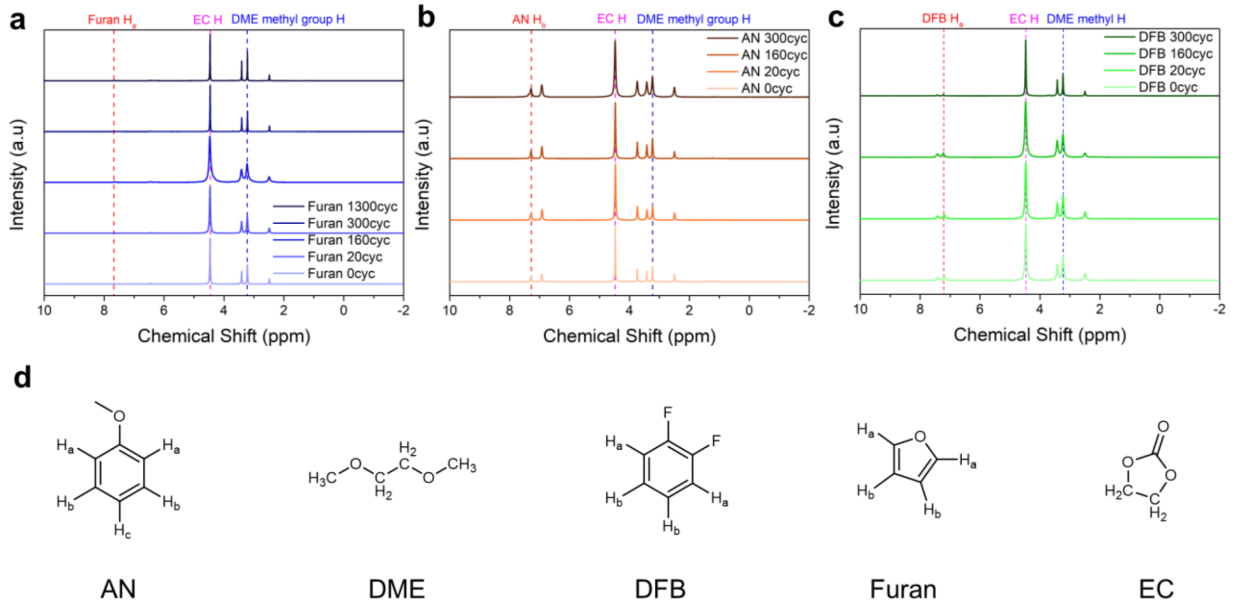

Supplementary Figure 42. ^{1}H-NMR spectra of electrolytes extracted from Li|Cu cells after different number of cycles. a 3 M_{solv} LiFSI DME:Furan-(1:2), **b** 3 M_{solv} LiFSI DME:AN-(1:2), and **c** 3 M_{solv} LiFSI DME:DFB-(1:2). **d** The molecular structures of each solvent used for NMR analysis. Protons are labelled with corresponding subscripts to indicate each NMR peaks. The peaks indicated are used for the quantitative analysis.

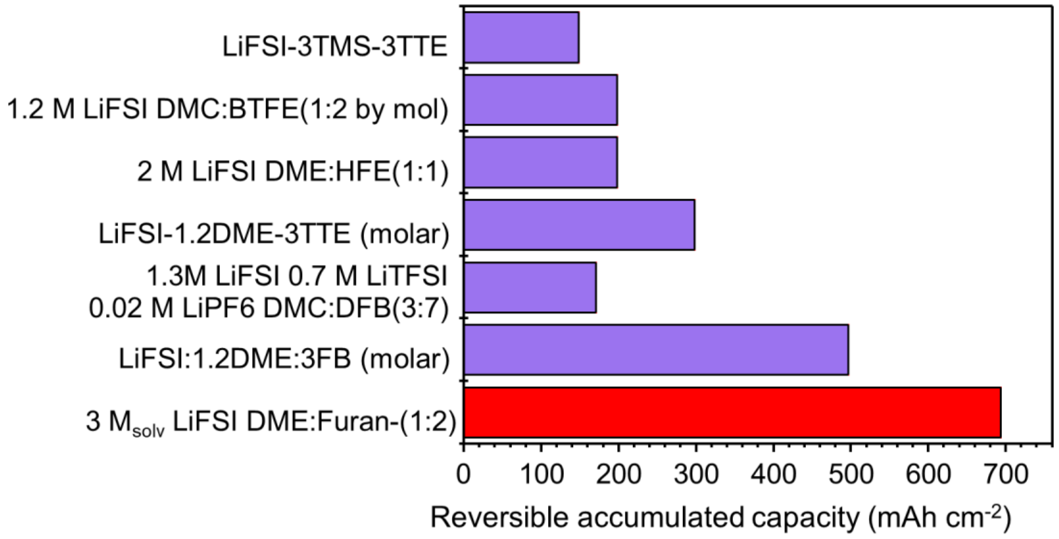

Supplementary Figure 43. Reversible accumulated capacity of electrolytes listed in Fig. 6a.

Molecule and Specific Configuration	Binding Energy (kJ per mol)	
	EC/DEC (1:1 v/v) Solvent	Vacuum
AN (binidng to oxygen atom)	-16.8	-177.2
AN (binidng to phenyl group)	-15.8	-181.9
MeCyHx (binding to oxygen atom)	-21.1	-195.6
BzMe (binidng to oxygen and front phenyl group)	-20.6	-224
Ethoxybenzene (binding to back or front phenyl group)	-14.9	-185.9
Ethoxybenzene (binding to oxygen atom)	-15.4	-183.6
THF (binding to oxygen atom)	-38	-201.6
Furan (binding to oxygen atom)	-0.5	-124.7
Furan (binding to front furan ring)	-5.4	-122.1

Supplementary Table 1. **Calculated binding energy of lithium ion with organic molecules under specific configuration.** The calculation was performed under EC/DEC (1:1v/v) solvent condition and vacuum condition.

Molecule	EC/DEC (1:1 v/v) Solvent		Vacuum	
	HOMO (eV)	LUMO (eV)	HOMO (eV)	LUMO (eV)
Furan	-6.529	-0.186	-6.461	-0.106
DME	-7.268	0.389	-7.136	0.35
AN	-6.87	-0.584	-6.748	-0.488
Ethoxybenzene	-6.844	-0.572	-6.703	-0.457
DFB	-7.156	-0.865	-7.169	-0.944

Supplementary Table 2. **HOMO and LUMO level of organic molecules studied in Fig. 5a.** The calculation was performed under EC/DEC (1:1 volume ratio) solvent condition and vacuum condition.

Solvent	Price ($ per L)
EC	110.1
DEC	200
DME	102
AN	68
FB	154.5
DFB	3416.4
Furan	116
Ethoxybenzene	110
TTE	2503.2
BTFE	41748
HFE	17458.4

Supplementary Table 3. **Price of solvents used in Fig. 6.** The origin of the data is TCI Chemicals USA website (https://www.tcichemicals.com/US/en/)

Supplementary References

1. Fang, C. *et al.* Pressure-tailored lithium deposition and dissolution in lithium metal batteries. *Nat Energy* **6**, 987–994 (2021).
2. Su, C.-C. *et al.* Solvating power series of electrolyte solvents for lithium batteries. *Energy Environ. Sci.* **12**, 1249–1254 (2019).
3. Kamlet, M. J. & Taft, R. W. The solvatochromic comparison method. I. The .beta.-scale of solvent hydrogen-bond acceptor (HBA) basicities. *J. Am. Chem. Soc.* **98**, 377–383 (1976).
4. Reichardt, C. Solvatochromic Dyes as Solvent Polarity Indicators. *Chem. Rev.* **94**, 2319–2358 (1994).
5. Yao, N. *et al.* An Atomic Insight into the Chemical Origin and Variation of Dielectric Constant in Liquid Electrolytes. *Angew. Chem. Int. Ed.* anie.202107657 (2021) doi:10.1002/anie.202107657.
6. Holoubek, J. *et al.* Tailoring electrolyte solvation for Li metal batteries cycled at ultra-low temperature. *Nat Energy* (2021) doi:10.1038/s41560-021-00783-z.
7. Thenuwara, A. C. *et al.* Efficient Low-Temperature Cycling of Lithium Metal Anodes by Tailoring the Solid-Electrolyte Interphase. *ACS Energy Lett.* **5**, 2411–2420 (2020).
